# Acupuncture ameliorates diet-induced obesity via the vagal–GLP-1–ARC circuit: neural mechanism of anorexigenic action

**DOI:** 10.1186/s13020-025-01274-z

**Published:** 2026-01-08

**Authors:** Yanan Yang, Yuwei Shao, Jun Tian, Yuezhu Wang, Ye Zhu, Siying Pan, Xiali Wei, Linyan Jiang, Xiaoke Wang, Qing Shu

**Affiliations:** 1 China Resources & Wisco General Hospital, Wuhan University of Science and Technology, Wuhan, China; 2 Department of Rehabilitation Medicine, Zhongnan Hospital of Wuhan University, No. 169 Donghu Road, Wuchang District, Wuhan, 430071 China; 3 College of Sports Medicine, Wuhan Sports University, Wuhan, China; 4 Hubei Province Key Laboratory of Occupational Hazard Identification and Control, Wuhan University of Science and Technology, Wuhan, China; 5 Department of Rehabilitation Medicine, Wuchang Hospital Affiliated to Wuhan University of Science and Technology, Wuhan, China

**Keywords:** Obesity, Electroacupuncture, Glucagon-like peptide-1, Vagus nerve, Appetite

## Abstract

**Background:**

Electroacupuncture (EA) has demonstrated efficacy in ameliorating obesity through its marked appetite-suppressing effects. This study aims to elucidate the peripheral-central communication mechanism underlying EA's appetite inhibition mediated by the “vagal afferent fiber–nucleus tractus solitarius (NTS)–hypothalamic arcuate nucleus (ARC)” neural circuit.

**Methods:**

High-fat diet-induced obese rats received EA or transcutaneous auricular vagus nerve stimulation (taVNS) for 8 weeks. Furthermore, we employed chemogenetic approaches to activate NTS glucagon-like peptide-1 (GLP-1) neurons (NTS^GLP−1^) and utilized capsaicin for gastric vagal deafferentation (GVND). Outcomes included metabolic profiles, vagal electrophysiology, expression of NTS^GLP−1^ and hypothalamic appetite-regulating neuropeptides, and neuronal activation markers.

**Results:**

We observed reduced expression of GLP-1 in the NTS of obese rats. Chemogenetic activation of NTS^GLP−1^ significantly suppressed appetite, mitigated obesity, and modulated hypothalamic pro-opiomelanocortin (POMC) and neuropeptide Y (NPY). Following EA intervention in obese rats, concurrent activation of NTS^GLP−1^ and vagal afferent fibers was observed. Similar to chemogenetic NTS^GLP−1^ activation, EA upregulated the anorexigenic peptide POMC while downregulating the orexigenic peptide NPY in the ARC. Chemogenetic inhibition of GLP-1 neurons during EA application partially inhibited its anti-obesity and anorectic effects. Further investigations revealed that both EA and taVNS effectively reduced food intake and alleviated obesity. While both interventions activated vagal pathways and NTS^GLP−1^, EA induced a significantly stronger activation of vagal afferent fibers compared to taVNS. Critically, GVND prior to EA application attenuated its anti-obesity effects.

**Conclusion:**

The therapeutic benefits of EA in appetite suppression and obesity mitigation are mediated by selective regulation of the “vagal–GLP-1–ARC” neural circuit.

**Graphical Abstracts:**

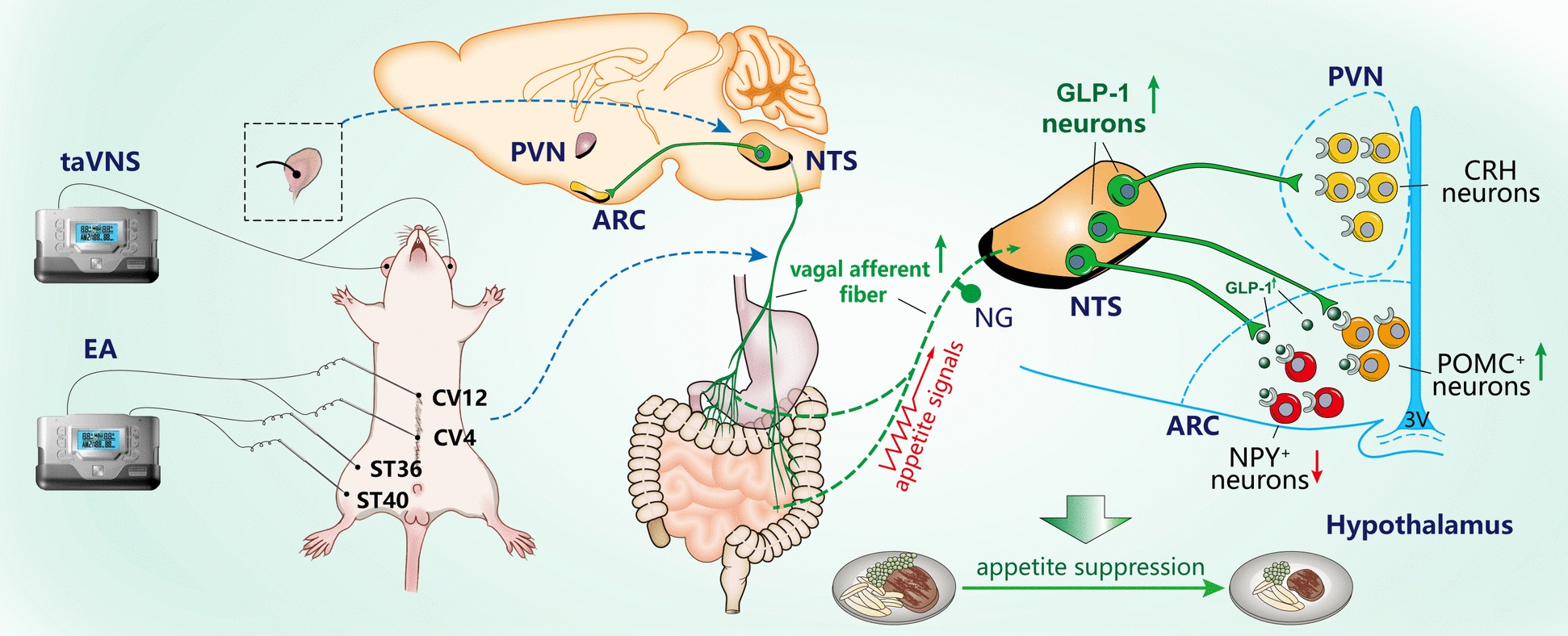

**Supplementary Information:**

The online version contains supplementary material available at 10.1186/s13020-025-01274-z.

## Introduction

The incidence of obesity has increased every year since 1980, the growing burden of obesity comprises a significant public health threat [1]. In 2019, Overweight and obesity were the sixth leading risk factor for death and disability [2]. Obesity increases the risk for developing multiple diseases, such as diabetes mellitus, cardiovascular disease [3], several types of cancers [4], an array of musculoskeletal disorders [5], even mood disorders [6]. Obesity-related personal medical cost and public health expenditures continue to rise in both developed and developing countries [7, 8].

Calorie restriction (CR) and exercise constitute cornerstone interventions for obesity management. Evidence confirms CR as a prerequisite for exercise-mediated improvements in obesity and type 2 diabetes [9]. Appetite regulation, involving multiple neural nuclei in the central nervous system, is crucial for obesity treatment [10]. Therapeutics targeting central neural circuits that induce appetite signal transduction have become a hot field [11]. Glucagon-like peptide-1 (GLP-1), a gut-derived peptide hormone, plays an important role in transmitting appetite signals from peripheral to central feeding-related nuclei via sympathetic and vagal pathways [12, 13]. Emerging studies highlight the central GLP-1 is also involved in energy metabolism, especially for appetite regulation [14]. It is produced by some non-catecholaminergic neurons in the caudal portion of the nucleus solitarius (NTS) [15], and these hindbrain preproglucagon (PPG) positive neurons are the primary source of endogenous brain that can synthesize GLP-1 [16]. This region is the termination nucleus of vagal afferent fibers, which can transmit appetite signals. Reduced vagus nerve responsiveness to orexigenic signals may contribute to obesity [12], while high frequency vagus nerve stimulation can improve obesity [17]. Physiologically, satiety signals activate NTS PPG neurons through vagal afferent fibers, increasing the synthesis and secretion of GLP-1 [18]. PPG neurons project to hypothalamus and release GLP-1 which bind to GLP-1 receptor (GLP-1R) in the appetite regulatory nucleus. This stimulates anorexigenic neurons like pro-opiomelanocortin (POMC) and corticotropin—releasing hormone in the arcuate nucleus (ARC) and paraventricular nucleus, suppressing food intake and improving obesity [19]. The neural circuit based on the vagal afferent fibers—NTS ^GLP−1^–ARC plays an important role in the central regulation of appetite.

Acupuncture, a traditional Chinese medical therapy authenticated by the WHO, is a rapidly growing complementary therapy [20, 21]. Evidence—based studies have shown its effectiveness in improving obesity [22–24]. The underlying mechanisms may be characterized by its multiple targets including suppressing appetite, regulating lipid metabolism, alleviating inflammation, promoting white adipose tissue (WAT) browning, inhibiting sympathetic nerve excitability, etc. [25–27]. People who receive acupuncture for weight loss experience a significant feeling of satiety after treatment [28]. Our previous research dissected how electroacupuncture (EA) modulates feeding behavior and ameliorates obesity, revealing its interaction with hypothalamic SIRT1 regulation and appetite-regulating peptide expression [29]. Notably, we further elucidated EA-induced GLP-1 activation in the NTS, which curbs excessive dopamine neuronal activity in obese rats, impeding reward-driven feeding [30]. Despite these advancements, the precise influence of EA, as a form of somatic surface stimulation therapy, on the central transmission of appetite signals from peripheral origins remains an enigma. Guided by these insights, the present study is poised to delineate the underlying mechanisms of EA in suppressing food intake and enhancing obesity management by potentially targeting the "vagal afferent fibers—NTS^GLP−1^—ARC" neural circuitry.

## Materials and methods

### Experimental animals and establishment of obesity model

This study utilized 8-week-old male Wistar rats (220 ± 20 g) from Beijing Vital River Laboratory Animal Technology Co. (SCXK(E)2020–0018), maintained under specific pathogen-free (SPF) environment (22 ± 2 °C, 50 ± 10% humidity, 12h light/dark cycle) at the Experimental Animal Center, Zhongnan Hospital of Wuhan University. Six rats were continuously fed a standard chow (3.8 kcal/g; 10% fat, 70% carbs, 20% protein) and assigned to normal diet control group (ND), while 60 rats received a high-fat diet (HFD, 5.5 kcal/g; 46.5% fat, 38.5% carbs, 15% protein) for 8 weeks to induce obesity [29]. After this induction period, 48 HFD-fed rats meeting the obesity criterion (≥ 20% higher body weight vs. ND group mean) were selected as validated models [30] and randomly divided into six subgroups (n = 8/group) to receive various types of intervention, including electroacupuncture, chemogenetic activation, chemogenetic inhibition, vagus nerve electrical stimulation, or vagus nerve blockade. All animal procedures were performed in accordance with the Declaration of Helsinki and approved by the Institutional Animal Care and Use Committee (IACUC) at Center for Animal Experiment, Wuhan University (No. WP2020-08085, Sup. 1).

### GLP-1 neuronal labeling and chemogenetic modulation

Based on our previous studies [30–32], we stereotaxically injected a cre-dependent recombinant adeno-associated virus (rAAV-GLP-1, BrainVTA Co., China) into the NTS (coordinates relative to lambda: AP =  − 3.2 mm, ML =  ± 0.5–0.7 mm, DV =  − 9.6 mm; 24° posterior tilt) and co-administered it with rAAV-Ef1a-DIO-EGFP (BrainVTA Co., China) at a 1:1 volumetric ratio (v/v) to achieve GFP labeling of GLP-1 neurons and their hypothalamic projections. After 3 weeks, immunofluorescence confirmed GFP specificity in GLP-1 neurons and axonal projections to hypothalamic nuclei.

For chemogenetic regulation, rAAV expressed dio and Designer Receptors Exclusively Activated by Designer Drugs (DREADDs), such as hM3Dq or hM4Di, were co-injected with rAAV-GLP-1. Neuronal activity was modulated via intraperitoneal clozapine-N-oxide (CNO, 1 mg/kg; Sigma-Aldrich). The effect of chemogenetic regulation has been validated in our previous study [31, 32]. All rAAVs were obtained from commercial companies (BrainVTA Co., Ltd, Wuhan, China), and corresponding sequence were showed in Table 1. All rats subjected to chemogenetic modulation received dual viral injections (rAAV-GLP-1 + DREADDs constructs). In other experimental groups (excluding those receiving vagal stimulation or blockade), only three rats per group were randomly selected for viral fluorescent labeling to verify targeting accuracy and projection patterns.

**Table 1:** Sequences of rAAV used in this study

rAAV	Sequence
rAAV-GLP-1	rAAV-GLP-CRE-flag-WPRE-pA
rAAV-GFP	rAAV-Efla-DIO-EGFP-WPRE-pA
rAAV-hM3D	rAAV-hSyn-DIO-hqM3D (Gq)-EGFP-WPRE-pA
rAAV-hM4D	rAAV-hSyn-DIO-hM4D (Gi)-EGFP-WPRE-pA

### Electroacupuncture procedure

The EA procedure in this experiment was consistent with previous studies. Rats were immobilized in specific rat suits. Stainless steel needles (0.30 × 15 mm, Suzhou Hua Tuo Medical Equipment Co., Ltd.) were inserted into the acupoints "Zu San Li (ST36) ", "Feng Long (ST40) ", "Guan Yuan (CV4) ", and "Zhong Wan (CV2) ". Paired electrodes from a Han's acupoint nerve stimulator (HANS LH202H, China) connected CV4/CV12 and ST36/ST40, delivering 2-Hz electrical stimulation at 1 mA for 10 min/session. The procedure was repeated three times a week for eight weeks. All groups underwent equivalent immobilization. To assess chemogenetic inhibition's impact on EA efficacy, rats expressing inhibitory hM4Di DREADDs in GLP-1 neurons received intraperitoneal CNO 30 min prior to EA.

### Transcutaneous auricular vagus nerve stimulation

The protocol of transcutaneous auricular vagus nerve stimulation (taVNS) were performed according to Rong, et al. [33]. Rats under 0.5–1% isoflurane anesthesia received bilateral auricular magnetic electrodes connected to a Han’s stimulator (HANS LH202H). Rats were mildly anesthetized using 0.5–1% isoflurane and received bilateral auricular magnetic electrodes connected to a Han’s stimulator (HANS LH202H). Parameters included 1-mA current, 2/15-Hz distant-dense wave alternating every 1 s. Mild auricular vibrations were employed to confirmed effective conduction. In the absence of vibration, saline swabs were addressed stimulation failures. This procedure was conducted once daily, with each session lasting for 30 min.

### Gastric vagus nerve deafferentation and in vivo detection of vagus nerve activity

In this study, capsaicin was used to chemically induce gastric vagus nerve deafferentation (GVND) in rats. Following a protocol from prior studies [34], an abdominal wall incision was made to access the stomach and surrounding structures. Both vagal trunks were visualized. Sterilized parafilm was placed under the posterior aspect of the stomach to exclude other abdominal viscera. A sterile gelatin sponge sling was gently secured around the gastro-esophageal junction. 0.1% capsaicin solution was prepared, capsaicin (1 mg) (404-86-4, aladdin, China) in 1 ml of vehicle (100 μl ethyl alcohol and 100 μl Tween80, and made up to 1 ml with 800 μl Saline). The solution was applied drop-wise to the gelatin sponge at 5-min intervals over 30 min, keeping the gelatin sponge throughout. After 30 min, the sponge and parafilm were removed, the cavity flushed with saline, and the incision sutured. To validate functional deafferentation, intragastric air inflation (5 ml, 30 s) was applied 30 min post-capsaicin exposure, while recording vagal afferent activity from the cervical vagus nerve.

For vagus nerve recording, anesthetized rats underwent cervical dissection to isolate the right cervical vagus nerve (~ 1 cm) alongside the carotid artery. The biological signal acquisition and processing system (MedLab-U/8c502, Nanjing, China) was connected to a computer. Its platinum hook electrode tip was secured and adjusted to hang 1–2 mm above the cervical vagus nerve. The nerve was carefully lifted to the hook without excessive tension. Two to three drops of pre—warmed sterile paraffin oil were applied to cover the nerve and incision, preventing dehydration. The Biomechanical Experiment software (MedLab V6.6.5) was used to observe and record nerve discharges. Only the right cervical vagus nerve was recorded for 10 min. The firing frequency, maximum voltage (Vmax), and effective voltage (Vrms) of the right cervical vagus nerve were collected to assess vagal afferent fiber activity.

### Body composition and feeding behavior analysis

Body mass and 24-h food intake were measured in all rats’ pre-intervention (week 0) and at 2-, 4-, 6-, and 8-weeks post-intervention. Lee’s index ( $\frac{\sqrt[3]{body mass \left(g\right)\times 1000}}{nasoanal length \left(cm\right)}$) was calculated and reported only at baseline (week 0) and endpoint (week 8) to assess longitudinal changes in adiposity status, with naso-anal length measured specifically at these two time points for this purpose. In a separate, acute behavioral experiment, food intake was monitored in HM3D-expressing obese rats at 0.5, 1, 2, 3, 15, and 24 h following intraperitoneal administration of CNO (1 mg/kg) to characterize the temporal dynamics of chemogenetic activation on feeding behavior. This acute monitoring was performed independently of the longitudinal 24-h intake measurements conducted at weeks 0–8. After euthanasia, abdominal, perirenal, and epididymal fat pads were dissected and weighed to determine body fat ratios.

### Hematoxylin and eosin staining

After transcardial perfusion, adipose tissues were fixed in 4% paraformaldehyde at 4 ℃ for 48 h, dehydrated, embedded in paraffin, and sectioned at 5μm. Sections were stained with Hematoxylin (blue—purple nuclei) and Eosin (pink cytoplasm and extracellular matrix). Lipid droplet areas in adipocytes were imaged via light microscopy (200 ×) and analyzed using ImageJ (v1.53).

### Detection of obesity-related plasma markers

After 8 weeks, 3 rats/group were anesthetized with isoflurane. Plasma was obtained via centrifugation after cardiac blood collection. Leptin and adiponectin levels were measured using an ELISA kit (cat. no. E-EL-R3012, E-EL-R0582c; Elabscience Biotechnology co., ltd.,). Two plasma samples/rat were used as biological replicates to minimize errors.

### Western blotting

Brain tissues (NTS and hypothalamus) were dissected from cervical dislocation-sacrificed rats (post-8-week intervention), flash-frozen in liquid nitrogen, and stored at − 80 °C. Tissues were processed using standard WB protocols. Primary antibodies included: rabbit anti-GLP-1 (1:1000, AF0166, Affinity), rabbit anti-POMC (1:1000, 22102–1-AP, Proteintech, China), rabbit anti-NPY (1:1000, 12833–1-AP, Proteintech, China). We used the housekeeping protein rabbit anti-β-actin (1:500, 20536–1-AP, Proteintech, China) and rabbit anti-GADPH (1:20,000, 10494–1-AP, Proteintech, China) for normalization. Triplicate WB membranes were imaged using an imaging system (SH-523, Shenhua, China). Protein expression (target/housekeeping OD ratios) was quantified via Gel-Pro Analyzer v4.0 (Polaroid Corporation, USA).

### Real-time polymerase chain reaction

Total RNA from NTS and hypothalamus brain tissues was extracted using Trizol® reagent, and concentrations were determined by the 260/280 nm absorbance ratio. 1 µg RNA was reverse-transcribed into cDNA using a HiScript II Q RT SuperMix kit (cat. no. R223-01; Vazyme Biotech Co., Ltd.). Gene expression of GLP-1, POMC, and NPY was quantified via SYBR—Green Real—Time PCR Master Mix Plus (Q111‑02; Vazyme Biotech Co., Ltd.), following the manufacturer’s protocols. Measurements were conducted in triplicate under standard reaction conditions and normalized to β-actin. Primers were obtained from TsingKe Biological Technology co., ltd. All temperature circulation and gene amplification were processed in a QuantStudio™ 6 real-Time PCR detection System (Thermo Fisher Scientific, Inc.). Primer sequences are in Table 2.

**Table 2:** Primer sequences for RT-PCR

Gene	Primer	Sequence (5' → 3')	PCR Products
β-actin	Forward	CACGATGGAGGGGCCGGACTCATC	240 bp
	Reverse	TAAAGACCTCTATGCCAACACAGT	
Rat GAPDH	Forward	AGGTCGGTGTGAACGGATTTG	123 bp
	Reverse	TGTAGACCATGTAGTTGAGGTCA	
Rat GLP-1	Forward	TCGTGGCTGGATTGTTT	142 bp
	Reverse	TGGCGTTTGTCTTCGTT	
Rat POMC	Forward	TGCCGAGATTCTGCTACAGTC	82 bp
	Reverse	CACCAGCTCCACACGTCTATG	
Rat NPY	Forward	CCGCTCTGCGACACTACATC	74 bp
	Reverse	AATCAGTGTCTCAGGGCTGGAT	

### Immunofluorescence

Rats’ Brain sections transduced with rAAV-mediated green fluorescent protein (GFP) underwent single-label immunofluorescence (IF) to stain POMC/NPY in ARC and c-fos in NTS. For non-GFP groups, double-label IF was performed to assess GLP-1/c-fos colocalization in NTS, and GLP-1R/POMC or GLP-1R/NPY in ARC to elucidate the regulatory relationship between GLP-1 neurons and the ARC. Rats were anesthetized with 2% pentobarbital (2 ml/kg, i.p.), perfused with 9% saline and 4% paraformaldehyde, post-fixed (4 °C, 72h), and dehydrated in graded sucrose (20–30%). Coronal sections (20 µm) were cut using a freezing microtome (CM1900, Lecia, Germany). After washed with TBST, sections were blocked with 10% goat/donkey serum (37 °C, 1h), incubated overnight at 4 °C with primary antibodies: rabbit anti-POMC (1:1000, ab210605, Abcam, USA), rabbit anti-NPY (1:1000, ab221145, Abcam, USA) or rabbit anti-c-fos (1:800, #2250, CST, USA), followed by secondary antibodies donkey anti-rabbit Alexa Fluor® 594 (1:500, ab150064, Abcam, USA) or goat anti-rabbit CoraLite594 (1:800, SA00013-4, Proteintech, China). For dual labeling, sections were re-blocked, incubated with mouse anti-GLP-1R (1:100, SC-390773, Santa Cruz, USA) or mouse anti-GLP-1 (1:200, #BF8043, Affinity, USA) and stained with secondary antibodies goat anti-mouse CoraLite488 (1:200, SA00013-1, Proteintech, China). Nuclei were counterstained with DAPI (Southern Biotech, China, 0100–20). Images were captured using a fluorescence microscope (BX53, Olympus). Four sections were randomly selected from each group for analysis. The ratio of positive cells to total cells or the integrated density of fluorescence in positive cells was determined as relative protein expression.

### Statistical analysis

Data are expressed as mean ± standard deviation ($\overline{x }\pm s$) and were analyzed using GraphPad Prism (version 9.4.1). Five rats per group that completed the full intervention protocol were included in obesity-related phenotyping analyses, accounting for unplanned discontinuations in some cohorts. For longitudinal measurements (body mass, food intake, Lee’s index), two-way repeated-measures ANOVA was applied to evaluate the main effects of time, group, and time × group interaction. If a significant interaction was detected (indicating group-dependent temporal divergence), post hoc multiple comparisons were conducted at individual time points, with the Sidak method for two-group comparison and the Tukey method for three-group comparison. Non-longitudinal data including relative protein/gene expression, plasma biomarkers, lipid droplet area, vagal electrophysiology, were analyzed using independent samples t-test for two-group comparisons and One-way ANOVA followed by Tukey’s post hoc test for three-group comparisons. Statistical significance was set at *P* < 0.05 (two-tailed).

## Results

### Obese rats exhibit marked hyperphagia accompanied by suppression of GLP-1 in NTS

**Fig. 1:**
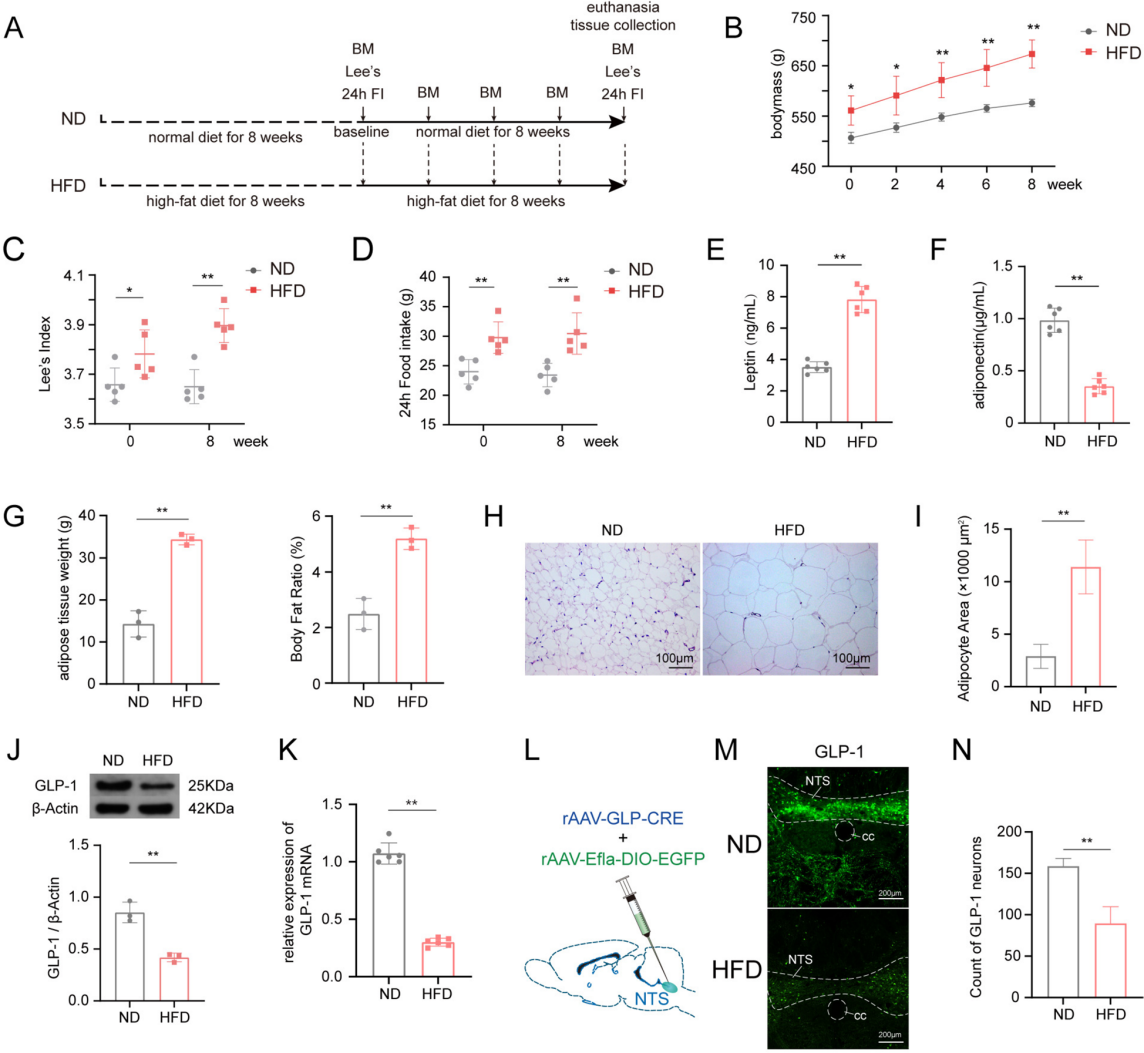
Obese rats exhibit hyperphagia accompanied by suppression of GLP-1 in NTS. **A** experimental timeline in this phase of the study; **B**–**D** comparison of the trends in bodymass gain, Lee's index and 24h food intake between normal and obese rats (n = 5/group); **E–F** comparison of plasma lipid metabolic biomarkers in normal versus obese rats; **G**–**I**, comparison of fat content between normal and obese Rats (H, 20 × objectives); **J**, **K**, protein and gene expression of GLP-1 in the NTS of normal and obese rats; **L** schematic diagram of viral strategy for labeling GLP-1 neurons in the NTS; **M** representative images of GLP-1 neurons in the NTS of normal and obese rats (10 × objectives); **N** quantitative analysis of GLP-1 neurons in the NTS of normal and obese rats. Compare to ND group, *P < 0.05, **P < 0.01. ND, normal diet control group; HFD, high-fat diet induced obese group; BM, bodymass; Lee ‘s, Lee's index; 24h FI, 24h food intake; GLP-1, glucagon-like peptide-1; NTS, nucleus solitarius; cc, central canal

To evaluate the successful establishment of the obesity model and further assess central GLP-1 expression levels in obese rats, we monitored phenotypic changes in these animals during continued administration of different diets post-model induction. As shown in Fig. 1, baseline measurements (Week 0) of body weight and Lee’s index showed significant differences between rats fed the ND and those fed the HFD, confirming successful induction of obesity. During the subsequent 8-week maintenance phase, HFD-induced obese rats exhibited significantly greater body weight gain and higher food intake compared to the ND-fed group (Fig. 1B–D). The leptin levels in the plasma of obese rats were higher than those in normal rats, whereas the levels of adiponectin were lower (Fig. 1E–F). Consequently, HFD-induced obese rats displayed significantly elevated body fat ratios and enlarged adipocytes compared to ND-fed controls (Fig. 1G–I). Additionally, the expression of GLP-1 protein and gene in the nucleus tractus solitarius (NTS) of obese rats was lower than that in normal rats (Fig. 1J, K). Following the injection of adeno-associated virus using the cre-loxp recombinase strategy, a reduction in the number of GLP-1 neurons in the NTS of obese rats was observed (Fig. 1L–N).

### Chemogenetic activation of GLP-1 neurons in NTS suppresses appetite and slows weight gain in obese rats

**Fig. 2:**
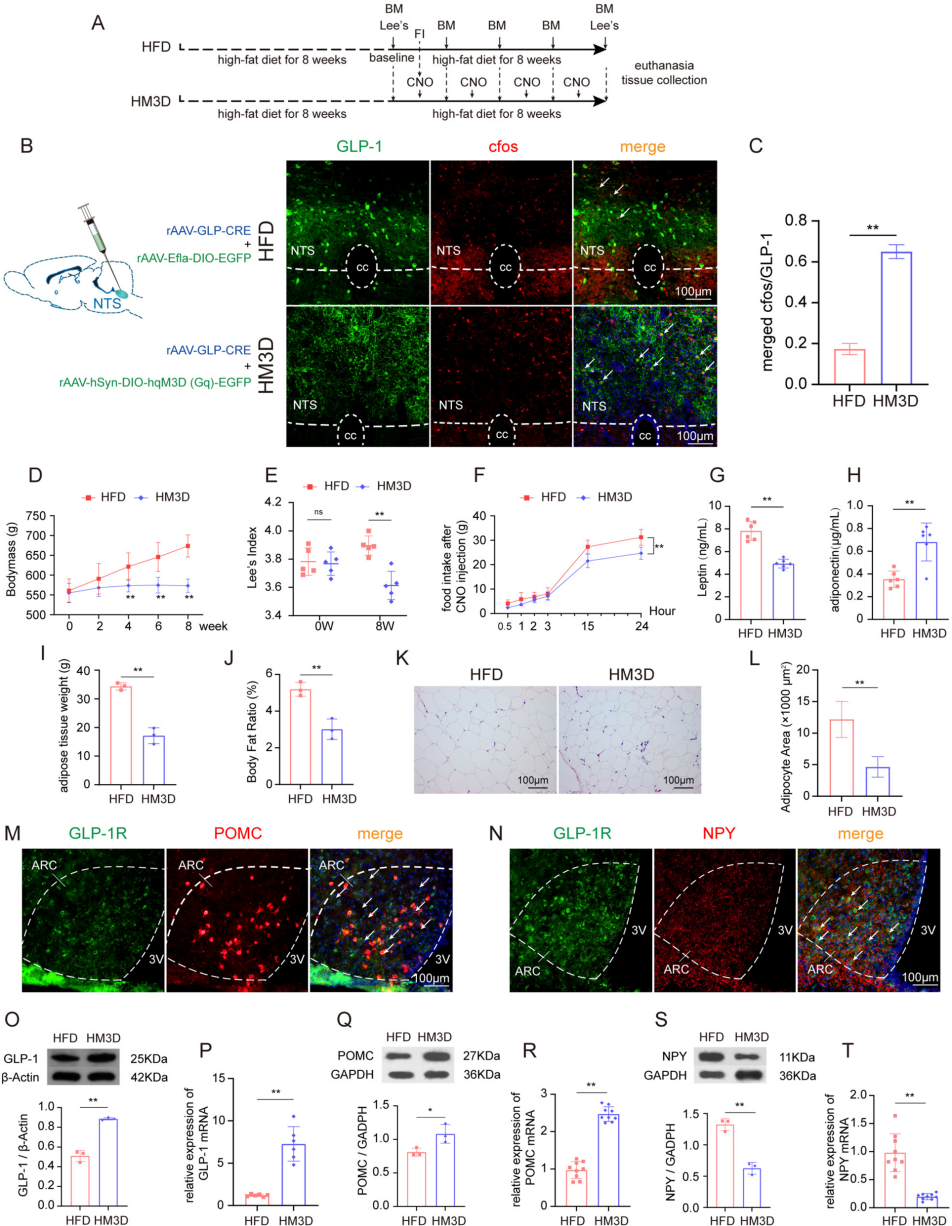
Activation of GLP-1 neurons can suppress appetite and slows weight gain in obese rats. **A** experimental timeline in this phase of the study; **B** schematic diagram of rAAV strategy for chemogenetic activate GLP-1 neurons in the NTS and representative images of GLP-1 neurons in the NTS of HFD group and HM3D group (20 × objectives); **C** quantitative analysis of activated GLP-1 neurons in the NTS of HFD group and HM3D group; **D**–**F** Trends in bodymass gain, Lee's index and 24h food intake after GLP-1 neurons activated (n = 5/group); **G**–**H** comparison of plasma lipid metabolic biomarkers between chemogenetic activated rats and obese rats; **I**–**L** comparison of fat content and adipocyte size between chemogenetic activated rats and obese rats (**K** 20 × objectives); **M**–**N** representative fluorescence double labeling images of GLP-1R/POMC and GLP-1R/NPY co-localization in the hypothalamic ARC (20 × objectives); **O**–**P**, comparison of protein and gene expression of GLP-1 in NTS Between chemogenetic activated rats and obese rats; **Q**–**T**, comparison of protein and gene expression of POMC and NPY in hypothalamus between chemogenetic activated rats and obese rats. Compare to HFD group, *P < 0.05, **P < 0.01. HFD, high-fat diet induced obese group; HM3D, high-fat diet induced obese group received HM3D activity; BM, bodymass; Lee ‘s, Lee's index; FI, food intake test; GLP-1, glucagon-like peptide-1; GLP-1R, glucagon-like peptide-1 receptor; POMC, pro-opiomelanocortin; NTS, nucleus solitarius; NPY, neuropeptide Y; ARC, arcuate nucleus; 3V, 3rd ventricle; cc, central canal

After fluorescently labeling and chemogenetic activating GLP-1 neurons in NTS of obese rats using rAAV, the GLP-1 in the NTS region of rats in the HM3D group was activated (Fig. 2B, C, Sup Fig. S2). The rapid increase in bodymass of obese rats was significantly suppressed, with the Lee’s index being lower than that in the obesity model rats after an 8-week intervention (Fig. 2D, E). Concurrently, we observed that after the activation of GLP-1 neurons, obese rats exhibited a significant reduction in food intake within 24 h (Fig. 2F). Following the 8-week intervention, the activation of GLP-1 neurons led to a decrease in leptin and an increase in adiponectin in plasma (Fig. 2G, H). Additionally, the activation of GLP-1 also reduced body fat content and adipocyte volume (Fig. 2I–L). Furthermore, we confirmed that in the hypothalamus, a downstream brain region of the GLP-1 neuron projection, neurons expressing orexigenic peptides POMC and NPY also expressed the GLP-1 receptor (Fig. 2M, N, Sup Fig. S2), indicating that these appetite-regulating neurons are regulated by GLP-1 neurons. Based on this, we observed that after the upregulation of GLP-1 in the NTS (Fig. 2O, P), the protein and gene expression of the anorexigenic peptide POMC in the hypothalamus increased (Fig. 2Q, R), while the expression of the orexigenic peptide NPY decreased (Fig. 2S, T).

### EA can suppress appetite and slow down weight gain in obese rats, accompanied by the activation of vagal afferent fibers and GLP-1 neurons in NTS

**Fig. 3:**
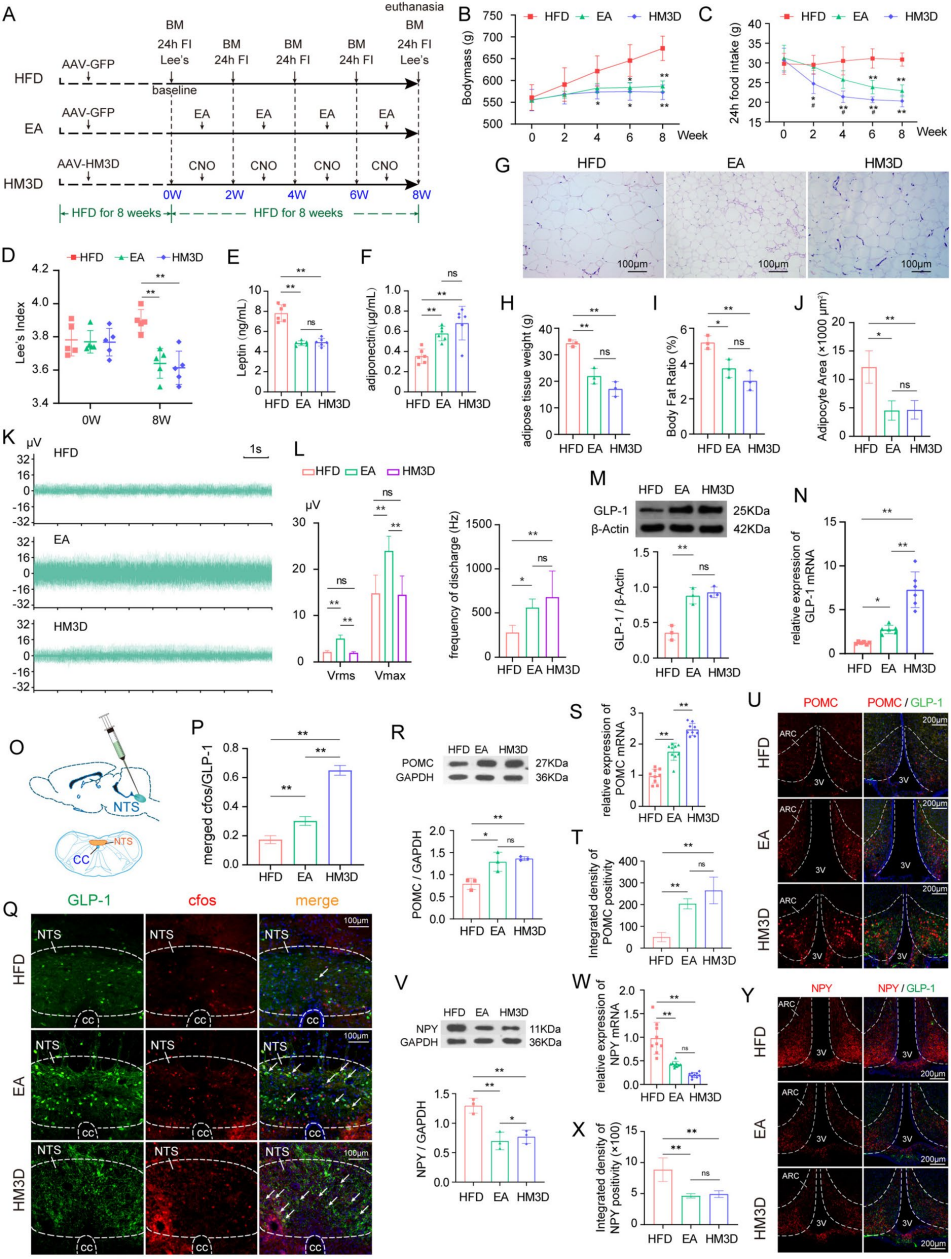
EA can improve obesity via activation of NTS^GLP−1^ and regulate POMC/NPY neuros in hypothalamus. **A** experimental timeline in this phase of the study; **B**–**D** comparative analysis of bodymass Gain, Lee's Index, and 24-h food intake among HFD, EA, and HM3D groups (n = 5/group); **E**–**F** comparison of plasma lipid metabolic biomarkers among HFD, EA, and HM3D groups; **G**–**J** comparison of fat content and adipocyte size among HFD, EA, and HM3D groups (**G** 20 × objectives); **K** representative image of right cervical vagus nerve discharge waveforms; **L** comparison of electrophysiological indices related to vagus nerve excitability among HFD, EA, and HM3D groups; **M**, **N**, protein and gene expression of GLP-1 in the NTS of model, EA, and HM3D groups; **O** schematic illustration of rAAV injection into the NTS and fluorescence localization; **P**, quantitative analysis of GLP-1 neurons in the NTS of HFD, EA, and HM3D groups; **Q** representative images of GLP-1 neurons in the NTS of HFD, EA, and HM3D groups (20 × objectives); **R**–**S** comparison of protein and gene expression of POMC in hypothalamus; **T**–**U** representative images and quantitative analysis of POMC-positive signaling in the ARC (10 × objectives); **V**–**W** comparison of protein and gene expression of NPY in hypothalamus; **X**–**Y**, representative images and quantitative analysis of NPY-positive signaling in the ARC (20 × objectives). Compare to HFD group, ^*^P < 0.05, ^**^P < 0.01; Compare to EA group, ^#^*P* < 0.05, ^##^*P* < 0.01. HFD, high-fat diet induced obese group; EA, obese rats received electroacupuncture; HM3D, high-fat diet induced obese rats received HM3D activity; BM, bodymass; Lee ‘s, Lee's index; 24h FI, total food intake in 24 h; GLP-1, glucagon-like peptide-1; POMC, pro-opiomelanocortin; NPY, neuropeptide Y; ARC, arcuate nucleus; 3V, 3rd ventricle; cc, central canal; V_max_, maximum voltage; V_rms_, effective voltage

As depicted in Fig. 3, prior to intervention, there were no statistically significant differences in bodymass, Lee's index, and 24-h food intake among obese rat models across different groups. During the EA treatment, the bodymass of obese rats also increased; however, the rate of gain was significantly slower compared to rats that did not receive intervention, exhibiting a statistical difference from the obese rats by the 6th week (Fig. 3B). The 24-h food intake also continued to decrease over the 8-week period, and it was significantly lower than that of the obese rats starting from the 6th week (Fig. 3C). This performance was consistent with the effect observed after chemogenetic activation of GLP-1 neurons, but the obese rats activated by chemogenetic showed a decrease in body weight and food intake earlier (Fig. 3B–D). Similarly, after EA or chemogenetic activation, the plasma leptin levels in rats decreased, and adiponectin levels increased (Fig. 3E, F), while fat content and adipocyte volume were reduced (Fig. 3G–J), reflecting the positive regulatory effect of the two intervention methods on lipid metabolism. Furthermore, although EA showed the same weight loss effect as GLP-1 activation, it was found to effectively activate the excitability of the vagal afferent fibers in obese rats (Fig. 3K, L), while the activation of GLP-1 neurons seemed to upregulate the firing frequency of the vagus nerve but did not significantly affect the maximum voltage (V_max_) and effective voltage (V_rms_). However, both intervention methods could upregulate the protein and gene expression levels of GLP-1 in the NTS region (Fig. 3M–Q, Sup Fig. S3). It is noteworthy that, following intervention via EA or chemogenetic activation, the nerve terminals of GLP-1 were found to be densely distributed in the ARC. In contrast, the distribution of these nerve terminals was relatively sparse in the model group. Immunofluorescence also showed that POMC neurons downstream of GLP-1 in the ARC were activated after EA, while NPY neurons were inhibited, similar to the performance of rats in the GLP-1 neuron activation group (Fig. 3T–U, X–Y, Sup Fig. S3). The protein and gene expression of POMC and NPY in the ARC region also showed the same trend in the three groups (Fig. 3R–S, V–W). This indicates that EA and the chemogenetic activation of GLP-1 neurons have similar mechanisms in regulating the “NTS^GLP−1^-ARC” circuit. However, the activating effects of EA on the NTS may be related to the activation of vagal afferent fibers.

### The anorexia effect of EA can be partially suppressed by chemogenetic inhibition of GLP-1.

**Fig. 4:**
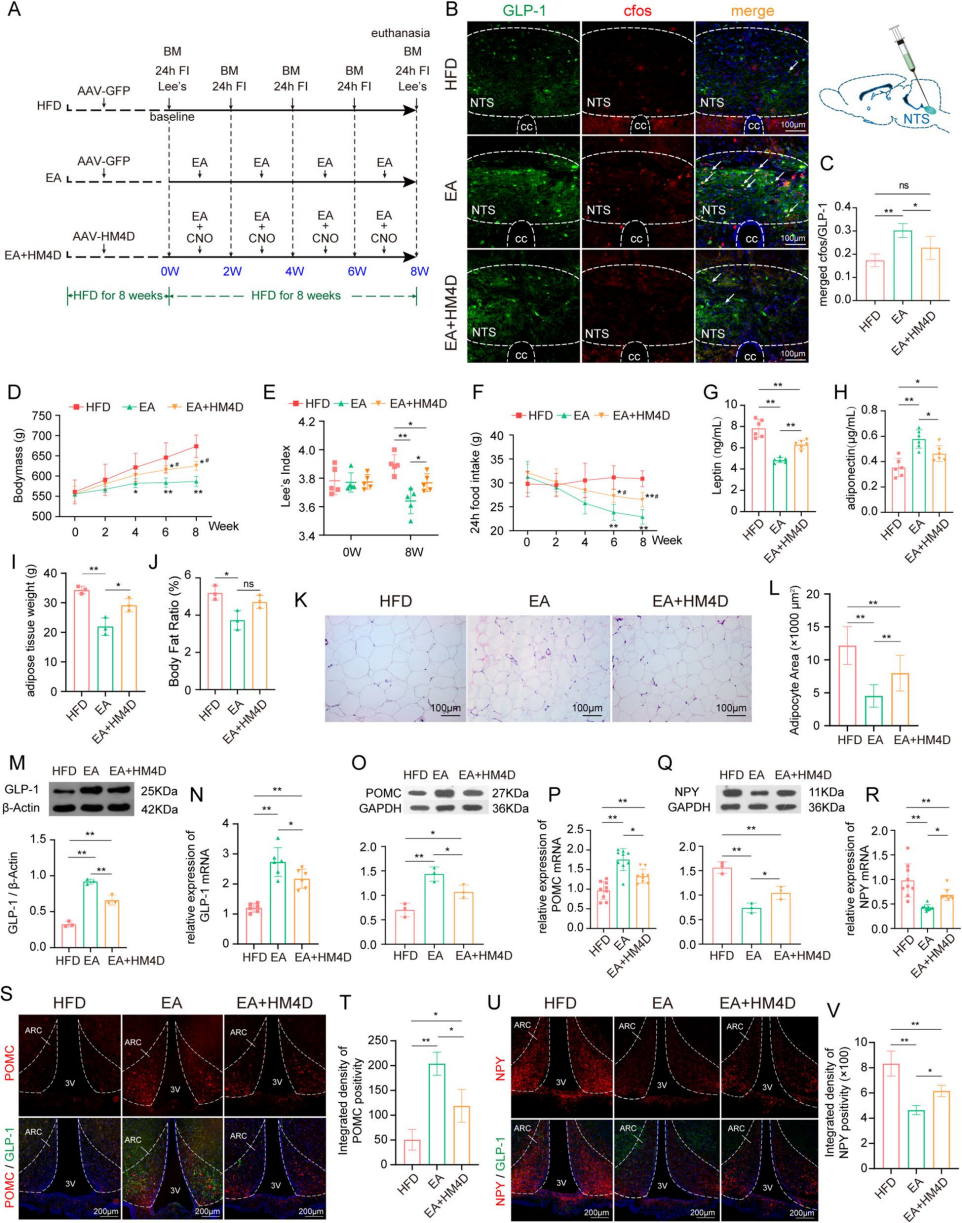
The anorexia effect of EA can be partially suppressed by chemogenetic inhibition of GLP-1. **A** experimental timeline in this phase of the study; **B** schematic diagram of rAAV strategy for chemogenetic activate GLP-1 neurons in the NTS and representative images of GLP-1 neurons among the HFD, EA, and EA + HM4D groups (20 × objectives); **C** quantitative analysis of activated GLP-1 neurons in the NTS of HFD, EA, and EA + HM4D group; **D**–**F** comparison of bodymass, Lee's index and 24h food intake (n = 5/group); **G**-**H**, comparison of plasma lipid metabolic biomarkers; **I**–**L** comparison of fat content and adipocyte size (**K** 20 × objectives); **M**, **N** comparison of protein and gene expression of GLP-1 in the NTS; **O**–**R** comparison of protein and gene expression of POMC and NPY in hypothalamus; **S**, **T** representative images and quantitative analysis of POMC-positive signaling in the ARC (10 × objectives); **U**, **V** representative images and quantitative analysis of NPY-positive signaling in the ARC (10 × objectives). Compare to HFD group, ^*^P < 0.05, ^**^P < 0.01; Compare to EA group, ^#^*P* < 0.05, ^##^*P* < 0.01. HFD, high-fat diet induced obese group; EA, obese rats received electroacupuncture; EA + HM4D, high-fat diet induced obese rats received both electroacupuncture and HM4D inhibition; BM, bodymass; Lee ‘s, Lee's index; 24h FI, total food intake in 24 h; GLP-1, glucagon-like peptide-1; POMC, pro-opiomelanocortin; NPY, neuropeptide Y; ARC, arcuate nucleus; 3V, 3rd ventricle; cc, central canal

Subsequently, we applied chemogenetic methods to suppress GLP-1 neurons during EA treatment to observe whether the effects of EA on suppressing food intake, improving obesity, and lipid metabolism were achieved through the specific activation of the "GLP-1—hypothalamus" pathway (Fig. 4A). Immunofluorescence results showed that EA effectively activated GLP-1 neurons in the NTS, and the number of activated GLP-1 neurons decreased significantly after chemogenetic suppression, demonstrating that the chemogenetic tools virus was functioning properly (Fig. 4B, C, Sup Fig. S4). As anticipated, during EA treatment, the suppression of GLP-1 neurons resulted in an increase in the rats' weight gain rate, Lee's index, and 24-h food intake; however, these measures remained lower than those of the untreated model group (Fig. 4D, F), indicating that the inhibition of GLP-1 was not sufficient to fully antagonize the effects of EA. We also observed the same trend in the levels of plasma leptin and adiponectin, which are related indicators of lipid metabolism (Fig. 4G, H). Although there were differences between the EA group and the EA combined with chemogenetic suppression group in terms of fat tissue weight and average adipocyte volume (Fig. 4I, K). There was no statistical difference in body fat percentage (Fig. 4J), and this negative result may be related to the sample size. Similarly, after the suppression of GLP-1, the effect of EA on upregulating the expression of POMC in the hypothalamus and downregulating the expression of NPY was partially reversed (Fig. 4M–V, Sup Fig. S4). The results suggest that the effects of EA in suppressing food intake, improving obesity, and lipid metabolism are associated with the specific activation of the "NTS^GLP−1^—ARC" neural circuit.

### EA and taVNS activated NTSGLP^−1^ through distinct pathways to improve obesity.

**Fig.5:**
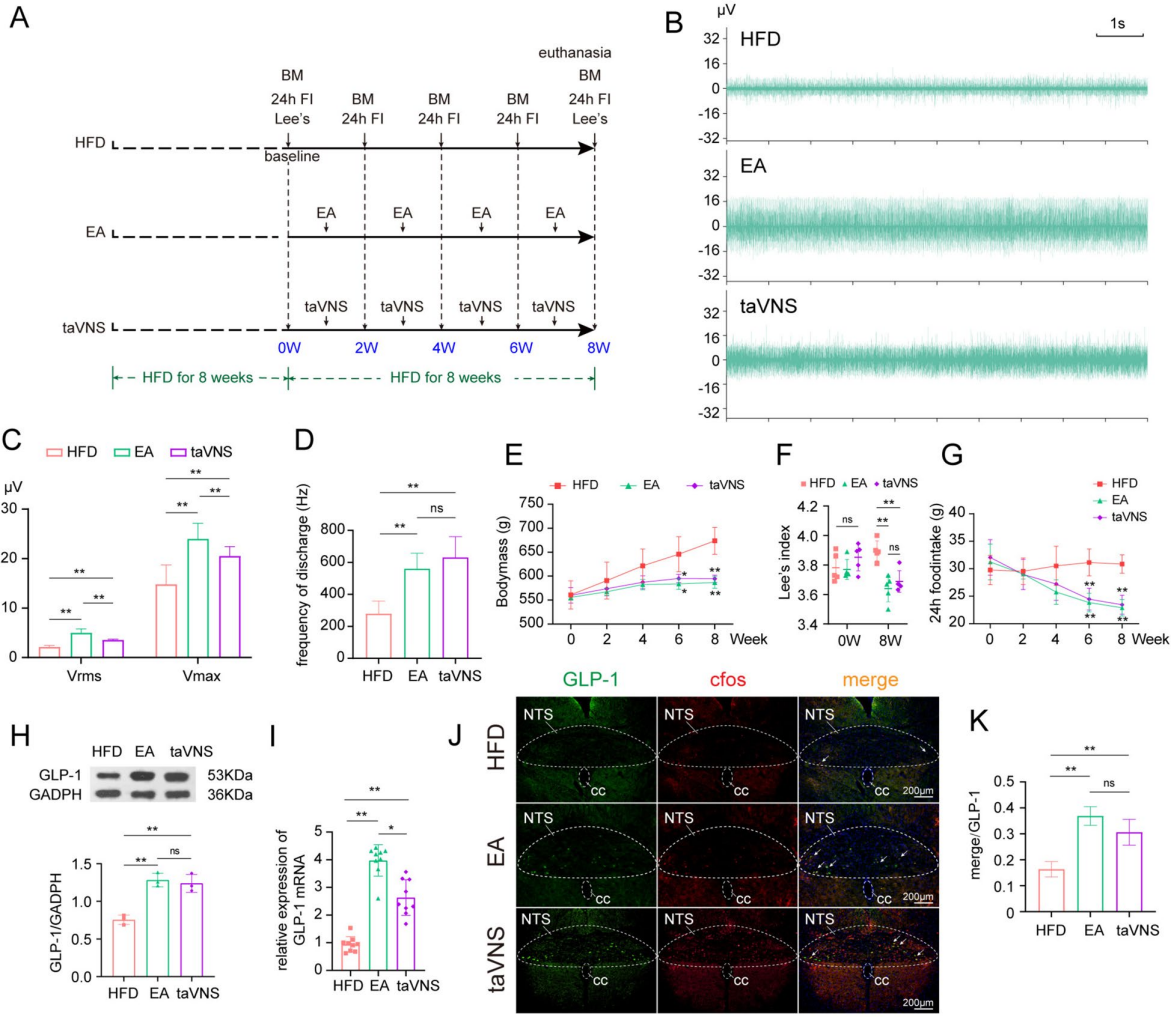
EA activated NTS^GLP−1^ by activating the afferent fibers of the vagus nerve, whereas taVNS did not. **A** experimental timeline in this phase of the study; **B** representative image of right cervical vagus nerve discharge waveforms of HFD, EA, and taVNS group; **C**, **D** comparison of electrophysiological indices related to vagus nerve excitability among HFD, EA, and taVNS group (n = 5/group); **E**–**G**, comparison of bodymass, Lee's index and 24 h food intake; **H**–**I**, comparison of protein and gene expression of GLP-1 in the NTS; **J**, representative images of GLP-1 neurons among the HFD, EA, and taVNS groups (10 × objectives); **K** quantitative analysis of activated GLP-1 neurons in the NTS. Compare to HFD group, ^*^P < 0.05, ^**^P < 0.01. HFD, high-fat diet induced obese group; EA, obese rats received electroacupuncture; taVNS, obese rats received transcutaneous auricular vagus nerve stimulation; BM, bodymass; Lee ‘s, Lee's index; 24h FI, total food intake in 24 h; GLP-1, glucagon-like peptide-1; NTS, nucleus solitarius; cc, central canal; V_max_, maximum voltage; V_rms_, effective voltage

In the aforementioned experiments, we observed that EA activated NTS^GLP−1^ and concurrently increased the excitability of vagal afferent fibers, indicating EA’s dual action on both central GLP-1 neurons and peripheral vagal inputs. To further clarify the pathway by which EA activates NTS^GLP−1^, we employed taVNS as a positive control to assess the comparative efficacy between EA and taVNS in addressing obesity and modulating the excitability of vagal afferent fibers (Fig. 5A). The findings indicated that both modalities were capable of enhancing the excitability of cervical vagal fibers, with EA demonstrating a more pronounced activating effect (Fig. 5B–D). This distinction may be attributed to the fact that the afferent pathway stimulated by auricular vagal nerve does not traverse the cervical vagus nerve. Nevertheless, both interventions effectively suppressed appetite and mitigated weight gain (Fig. 5E–G), and importantly, both activated NTS^GLP−1^ (Fig. 5H–K, Sup Fig. S5). The results indicate that EA and taVNS have similar effects in improving obesity and both activate NTS^GLP−1^; however, the pathways of action differ, with the weight loss effect of EA being closely associated with the activation of gastric vagal afferent fibers.

### Gastric vagus nerve deafferentation suppresses the effects of EA in activating NTSGLP^−1^ and improving obesity.

**Fig.6:**
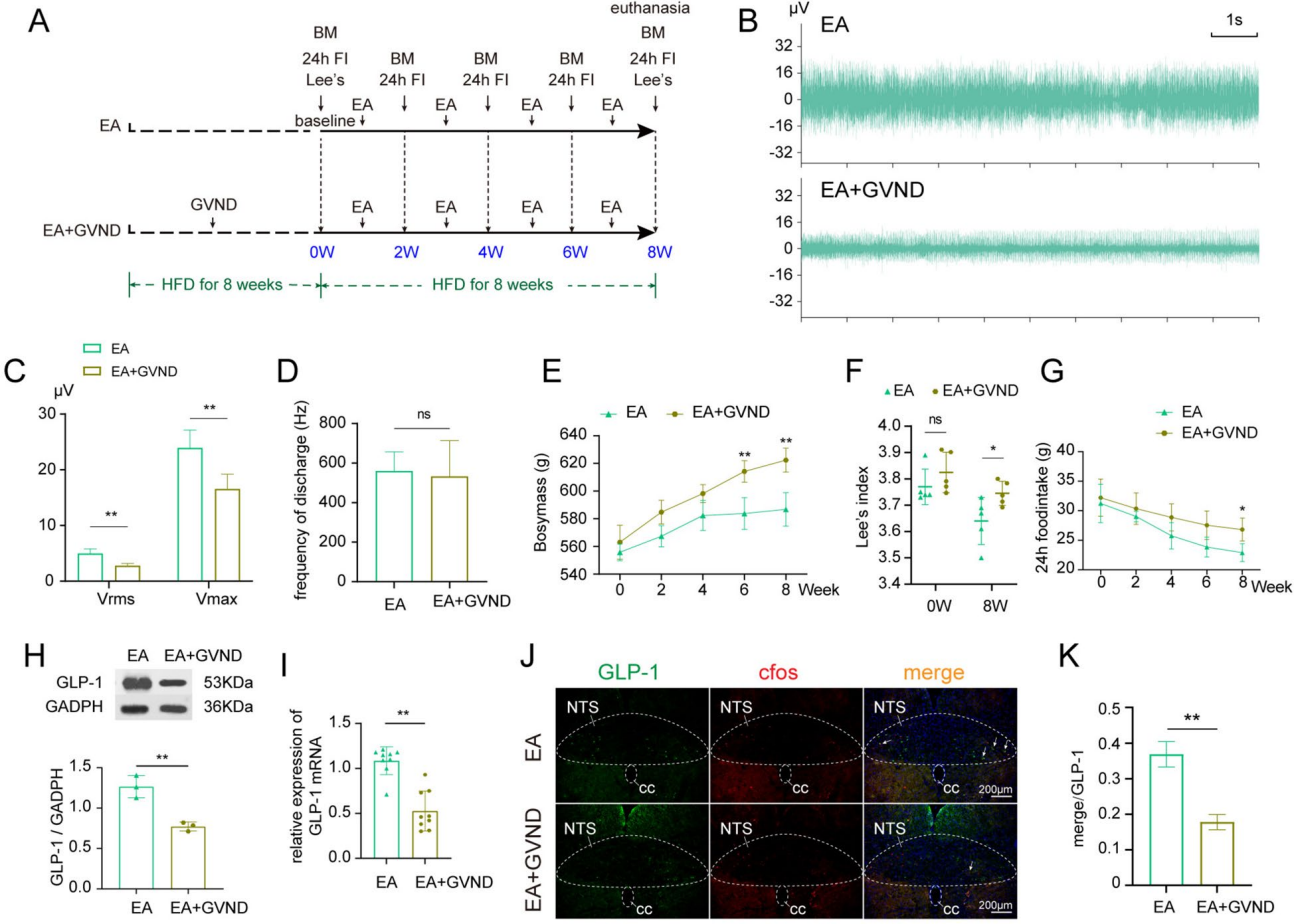
Gastric vagus nerve deafferentation partially suppresses the effects of EA in improving obesity. **A** experimental timeline in this phase of the study; **B** representative image of right cervical vagus nerve discharge waveforms of EA and EA + GVND group; **C**, **D** comparison of electrophysiological indices related to vagus nerve excitability between EA and EA + GVND group; **E**–**G** comparison of bodymass, Lee's index and 24h food intake of EA and EA + GVND group (n = 5/group); **H**, **I** comparison of protein and gene expression of GLP-1 in the NTS; **J** representative images of GLP-1 neurons between EA and EA + GVND group (10 × objectives); **K** quantitative analysis of activated GLP-1 neurons in the NTS. Compare to EA group, ^*^P < 0.05, ^**^P < 0.01. EA, obese rats received electroacupuncture; EA + GVND, high-fat diet induced obese rats received gastric vagus nerve deafferentation before electroacupuncture treatment; GVND, gastric vagus nerve deafferentation; BM, bodymass; Lee ‘s, Lee's index; 24 h FI, total food intake in 24 h; GLP-1, glucagon-like peptide-1; NTS, nucleus solitarius; cc, central canal; V_max_, maximum voltage; V_rms_, effective voltage

To further delineate the modulatory influence of EA on the "NTS^GLP−1^-ARC" neural circuit and ascertain whether this effect is specifically mediated through the excitation of gastric vagal afferent fibers, we implemented GVND utilizing capsaicin prior to EA intervention (Fig. 6A). The findings revealed a significant reduction in both the V_max_ and V_rms_ of the cervical vagus nerve in rats subjected to the combination of EA and GVND, in contrast to the EA group alone (Fig. 6B, C). Notably, the alterations in the discharge frequency were observed to be non-significant (Fig. 6D). Concurrently, rats that underwent GVND exhibited a pronounced increase in body weight gain, Lee's index, and 24-h food intake compared to the EA group (Fig. 6E–G). Furthermore, the expression of GLP-1 within the NTS was found to be downregulated in rats concurrently treated with vagal deafferentation and EA (Fig. 6H–K, Sup Fig. S6). These outcomes underscore the intricate relationship between the neural circuitry involving “vagal afferent fibers—NTS^GLP−1^—ARC” and the mechanisms by which EA exerts its anorectic, obesity-ameliorating, and lipid metabolism-modulating effects.

## Discussion

The development of obesity is influenced by a multitude of aspect, including genetic, biochemical, psychological, environmental, behavioral, and socio-demographic factors, with overeating being a major contributor [35]. There were two classical pathways involved in the transduction of appetite signaling from the periphery to the central nervous system (CNS), including adiposity signals and satiety signals [36]. Circulating metabolic hormones, including leptin, insulin, adiponectin, and ghrelin, are transmitted from the periphery to the ARC in hypothalamus by passing across the median eminence, regulating long-term energy metabolism [37]. Satiety signals is produced by mechanical sense of gastric mechanoreceptor and nutrient sensation of mucosal entero‐endocrine cells [38]. When food is ingested, it stimulates the expansion of stomach and activation of endocrine cells in small intestine, which activates vagal afferent fibers. These fibers transmit signals to brainstem, relaying information to various brain regions, including the hypothalamus [36]. Additionally, impaired vagal function had also been observed in obesity rodent [39, 40]. As the key termination nuclei of vagal afferent fibers, the NTS serves as a relay station for vagal-mediated satiety signals to the hypothalamus [41]. GLP-1 is a key gut-derived hormone that plays a crucial role in satiety signaling, which were synthesized both in enteroendocrine L cells and neurons in NTS [42]. Building on recent evidence that GLP-1 mediates state-dependent synaptic regulation in descending hypothalamic-brainstem circuits to control feeding and energy homeostasis [43]. In this present study, obese rats showed a markable increasing in appetite accompanied by a reduced level of activation in the NTS^GLP−1^ and a downregulation of GLP-1 protein and gene expression. Targeting NTS^GLP−1^ to explore its mechanism in improving obesity while evaluating its clinical efficacy would be worthwhile.

Evidence-based medical results had been accumulated for treating obesity by administrated by targeting peripheral GLP-1. Semaglutide, a novel GLP-1 analogue, were proved to be effective in treating 2DM and decreasing bodyweight [44, 45], owing to its marked anorexigenic effect [46]. However, some adverse reactions such as gastrointestinal disorders, infections, and metabolism/nutrition disorders challenge GLP—1 agonists' application [47]. Echoing the findings from our prior research [30], the current study employed chemogenetic activation of NTS^GLP−1^, which resulted in a pronounced suppression of appetite, effectively stalling weight gain in rats. Furthermore, we observed that the anorectic impact of an intraperitoneal CNO injection in obese rats persisted beyond 24 h. Our earlier studies had indicated that the appetite-suppressing effect of CNO in normal rats could extend to 48 h [32]. These findings highlight GLP—1 activation's strong inhibitory impact on feeding behavior. As energy intake diminishes, the associated disruptions in energy balance and lipid metabolism were rectified.

The reduction in vagal excitation mediated by high-fat-diet is one of the important reasons hindering the transmission of satiety signals [48, 49]. An increasing number of clinical studies and animal experiments have demonstrated the role of invasive vagus nerve stimulation in inducing satiety, improving obesity, and energy metabolism. However, its development is limited by the need for surgery, lack of standardized methods, and low—level evidence [50]. The development of taVNS in recent years has provided a non-invasive neuromodulation method that differs from previous methods requiring implantation of electrodes in the neck or diaphragm [51, 52]. taVNS activates the vagus nerve by electrically stimulating the auricular branch of the vagus nerve, and existing studies have confirmed it’s improvement on postprandial blood glucose in patients with impaired glucose tolerance [53]. The latest research further confirms that taVNS slows gastric motility by inducing vagal efferent impulses, highlighting it regulates appetite through the dorsal vagal complex [54]. In addition, evidence proving the regulatory effect of taVNS on the hypothalamus, the central regulator of energy [55]. Our investigation has substantiated the therapeutic potential of taVNS in mitigating food consumption, ameliorating adiposity, and modulating energy metabolism in rodent models. Notably, our findings suggest that the activation of NTS^GLP−1^ by taVNS could constitute an intrinsic mechanism underlying its therapeutic efficacy. Nonetheless, our research has observed taVNS had minimal impact on cervical vagus nerve voltage but significantly affected the NTS, likely attributed to the fact that the targeted auricular branch does not traverse the cervical pathway.

Existing studies has substantiated the affirmative impact of acupuncture on obesity [56], and its regulatory effects on the vagus nerve have also been validated through various clinical studies [57, 58] and animal experiments [59, 60], with acupoints such as ST36 (Zusanli) being highlighted [61]. Although our previous research has explored the preliminary mechanisms of EA in the hypothalamus SIRT1-mediated homeostatic feeding and the NTS^GLP−1^-mediated reward feeding [29, 30], the specificity related to the functional connectivity between neural nuclei and the EA effect has not been deeply probed. Moreover, the existing literature does not yet report on the relationship between the regulatory impact of EA on the two central appetite centers and its influence on the excitability of the afferent vagus nerve. Expanding upon these findings, the present study has provided further validation that chemogenetic activation of GLP-1 neurons can stimulate the activity of POMC within the hypothalamus while concurrently suppressing NPY. This dual action results in the dampening of appetite, contributing to the amelioration of obesity. Subsequently, we further confirmed that both EA and taVNS have an excitatory effect on the afferent fibers of the vagus nerve, thereby upregulating the NTS^GLP−1^ to exert the effect of suppressing appetite and improving obesity. In addition, we have specifically targeted and blocked the pivotal elements responsible for the EA effect, the GLP-1 neurons and the afferent fibers of the vagus nerve, and found that the effect of EA is partially antagonized. This finding underscores the specificity of the neural circuit regulation of "vagal afferent fibers—NTS^GLP−1^—ARC" by EA, and it also further enriches the multi-target mechanism of EA in improving obesity.

This study has several acknowledged limitations. Although low dose capsaicin was applied to selectively ablate gastric vagal afferents, a method well established in pharmacology, the overall toolkit for targeted vagal afferent manipulation remains limited [62]. We performed comprehensive obesity phenotyping, including measurements of body weight, Lee’s index, lipid metabolic profiles, and histopathological analysis of adipose tissue. However, due to limited access to specialized equipment, we were unable to perform indirect calorimetry or infrared thermography, methodologies that would have permitted a more in-depth characterization of energy metabolism. While our immunofluorescence data and neural fiber labeling support a functional connection between the NTS and ARC, definitive monosynaptic tracing using transsynaptic viral tools in genetically defined cre lines remains technically demanding and was not feasible in this phase of the study. Furthermore, while GLP 1 serves as a key mediator, its interactions with other gut derived signals such as CCK and ghrelin, as well as broader effects on thermogenesis and energy expenditure, require dedicated investigation. These aspects are recognized as essential directions for future research.

## Conclusion

In summary, this study further establishes that the modulation of vagal afferent fibers, which are integral to the transmission of satiety signals, constitutes one of the pivotal pathways through which EA exerts its appetite-regulating effects. It elucidates the mechanism by which EA, via the activation of vagal afferent fibers, can upregulate NTS^GLP−1^, consequently influencing the levels of hypothalamic appetite peptides POMC and NPY. Ultimately, it provides a comprehensive demonstration of the “vagal afferent fibers—NTS^GLP−1^—ARC” neural circuit related to appetite, which plays a role in the mechanism of EA's suppression of feeding, improvement of obesity, and modulation of lipid metabolism. The findings offer significant experimental evidence supporting the therapeutic use of acupuncture in obesity and in reducing the risk of cardiovascular diseases, type 2 diabetes, and non-alcoholic fatty liver disease. Future research should delve deeper into the interaction between central and peripheral energy metabolism organs, with a focus on appetite regulation as an entry point, to further explore the neural circuits in acupuncture studies.

## Supplementary Information

**Additional file 1.**

**Additional file 2.**

**Additional file 3.**

**Additional file 4.**

**Additional file 5.**

**Additional file 6.**

**Additional file 7.**

**Additional file 8.**

**Additional file 9.**

## Data Availability

Most raw data, including but not limited to original representative microscopy images at various magnifications, uncropped and unprocessed Western blot bands, and all associated quantitative datasets, have been provided in the Supplementary Materials to ensure full transparency and reproducibility. The other data that support the findings of this study are available from the corresponding author upon reasonable request.

## Abbreviations

ARC: Arcuate nucleus
CNO: Clozapine-N-oxide
EA: Electroacupuncture
GLP-1: Glucagon-like peptide-1
GLP-1R: GLP-1 receptor
GVND: Gastric vagal deafferentation
NTS: Nucleus tractus solitarius
taVNS: Transcutaneous auricular vagus nerve stimulation
POMC: Pro-opiomelanocortin
NPY: Neuropeptide Y
Vmax: Maximum voltage
Vrms: Effective voltage
ANOVA: Analysis of variance
