# Supplementary material for: Acupuncture ameliorates diet-induced obesity via the vagal–GLP-1–ARC circuit: neural mechanism of anorexigenic action
Source: Chin Med. 2026 Jan 8;21:20. doi: 10.1186/s13020-025-01274-z (PMC12781781; doi:10.1186/s13020-025-01274-z)
Supplement: Supplementary file 2 — Additional file 2. [file 13020_2025_1274_MOESM2_ESM.pdf]

Raw data of immunofluorescence experiments

Table 1.   Fluorescence Quantification of GLP-1 in figure 2

	GLP-1 positive cells	cfos/GLP-1 co-positive cells	ratio
HFD1	63	10	0.15873
HFD 2	72	15	0.208333
HFD 3	72	13	0.180556
HFD 4	48	7	0.145833
HM3D1	81	53	0.654321
HM3D2	92	64	0.695652
HM3D3	87	54	0.62069
HM3D4	89	56	0.629213

Table 2. Fluorescence Quantification of GLP-1, POMC, and NPY in figure 3

	GLP-1 positive cells	cfos/GLP-1 co-positive cells	ratio
HFD1	63	10	0.15873
HFD 2	72	15	0.208333
HFD 3	72	13	0.180556
HFD 4	48	7	0.145833
EA1	104	30	0.288462
EA2	94	30	0.319149
EA3	60	16	0.266667
EA4	117	39	0.333333
HM3D1	81	53	0.654321
HM3D2	92	64	0.695652
HM3D3	87	54	0.62069
HM3D4	89	56	0.629213
Integrated density of POMC fluorescence			
	**Area**	**Mean**	**IntDen**
HFD1	2.005	38.527	77.233
HFD 2	1.282	35.123	45.028
HFD 3	1.181	44.809	52.919
HFD 4	0.684	39.33	26.920
EA1	6.102	38.336	233.944
EA2	8.515	23.317	198.538
EA3	5.826	30.47	177.506
EA4	7.815	26.317	205.667
HM3D1	4.457	54.904	244.691
HM3D2	6.257	56.15	351.349
HM3D3	7.211	28.651	206.593
HM3D4	5.257	49.15	258.381
Integrated density of NPY fluorescence			
	**Area**	**Mean**	**IntDen**
HFD1	14.863	47.322	703.331
HFD 2	17.78	47.884	851.381
HFD 3	22.075	52.293	1154.354
HFD 4	17.2	48.293	830.639
EA1	9.412	44.705	420.764
EA2	10.527	44.194	465.22
EA3	10.356	44.654	462.444
EA4	11.356	44.654	507.091
HM3D1	10.17	44.825	455.881
HM3D2	11.002	44.783	492.705
HM3D3	10.361	43.687	452.632
HM3D4	12.687	44.689	566.983

Table 3. Fluorescence Quantification of GLP-1, POMC, and NPY in figure 4

	GLP-1 positive cells	cfos/GLP-1 co-positive cells	ratio
HFD1	63	10	0.15873
HFD 2	72	15	0.208333
HFD 3	72	13	0.180556
HFD 4	48	7	0.145833
EA1	104	30	0.288462
EA2	94	30	0.319149
EA3	60	16	0.266667
EA4	117	39	0.333333
EA+HM4D1	32	8	0.25
EA+HM4D2	35	10	0.285714
EA+HM4D3	40	7	0.175
EA+HM4D4	40	8	0.2
Integrated density of POMC fluorescence			
	**Area**	**Mean**	**IntDen**
HFD1	2.005	38.527	77.233
HFD 2	1.282	35.123	45.028
HFD 3	1.181	44.809	52.919
HFD 4	0.684	39.33	26.920
EA1	6.102	38.336	233.944
EA2	8.515	23.317	198.538
EA3	5.826	30.47	177.506
EA4	7.815	26.317	205.667
EA+HM4D1	3.178	38.923	123.688
EA+HM4D2	3.497	44.753	156.518
EA+HM4D3	1.786	42.642	76.164
EA+HM4D4	2.786	42.642	118.801
Integrated density of NPY fluorescence			
	**Area**	**Mean**	**IntDen**
HFD1	14.863	47.322	703.331
HFD 2	17.78	47.884	851.381
HFD 3	22.075	52.293	1154.354
HFD 4	17.2	48.293	830.639
EA1	9.412	44.705	420.764
EA2	10.527	44.194	465.22
EA3	10.356	44.654	462.444
EA4	11.356	44.654	507.091
EA+HM4D1	16	41.514	664.224
EA+HM4D2	13.7	43.006	589.1822
EA+HM4D3	14.1	40.315	568.4415
EA+HM4D4	14.105	45.486	641.58

Table 4. Fluorescence Quantification of GLP-1 in figure 5

	GLP-1 positive cells	cfos/GLP-1 co-positive cells	ratio
M5	13	2	0.153846
M6	29	6	0.206897
M7	21	3	0.142857
M8	20	3	0.15
EA5	32	11	0.34375
EA6	19	7	0.368421
EA7	31	13	0.419355
EA8	35	12	0.342857
taVNS1	32	11	0.34375
taVNS2	21	5	0.238095
taVNS3	40	12	0.3
taVNS4	38	13	0.342105

Table 5. Fluorescence Quantification of GLP-1 in figure 6

	GLP-1 positive cells	cfos/GLP-1 co-positive cells	ratio
EA5	32	11	0.34375
EA6	19	7	0.368421
EA7	31	13	0.419355
EA8	35	12	0.342857
EA+GVND1	23	4	0.173913
EA+GVND2	20	3	0.15
EA+GVND3	30	6	0.2
EA+GVND4	32	6	0.1875
