# Supplementary material for: Acupuncture ameliorates diet-induced obesity via the vagal–GLP-1–ARC circuit: neural mechanism of anorexigenic action
Source: Chin Med. 2026 Jan 8;21:20. doi: 10.1186/s13020-025-01274-z (PMC12781781; doi:10.1186/s13020-025-01274-z)
Supplement: Supplementary file 3 — Additional file 3. [file 13020_2025_1274_MOESM3_ESM.pdf]

## Raw gel images of GLP-1 in figure 1

GLP-1 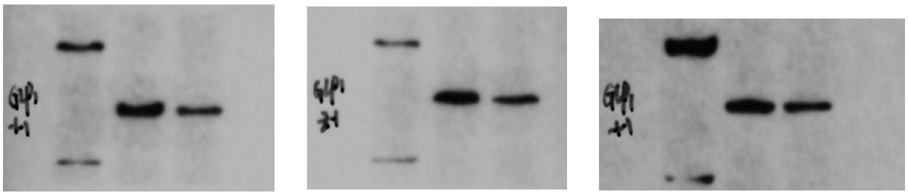

β-actin 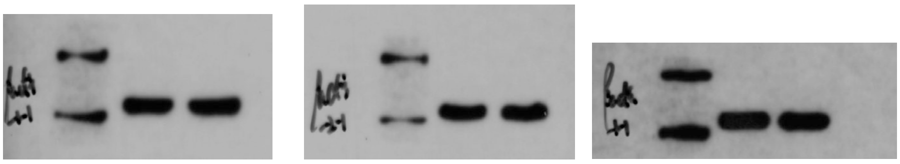

Quantification

| ND | HFD |
|---|---|
| 0.9641 | 0.3718 |
| 0.8194 | 0.4409 |
| 0.7741 | 0.4469 |

## Raw gel images of GLP-1 in figure 2

GLP-1 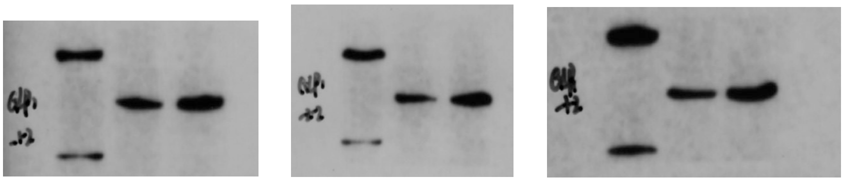

β-actin 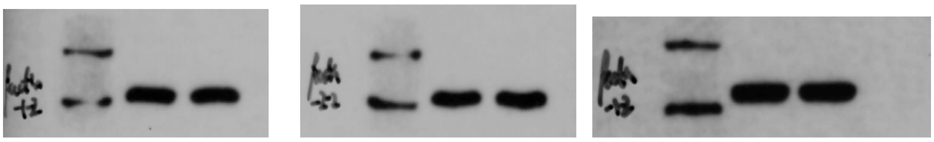

Quantification

| HFD | HM3D |
|---|---|
| 0.4439 | 0.8748 |
| 0.5476 | 0.8866 |
| 0.5366 | 0.8993 |

**Raw gel images of POMC and NPY in figure 2**

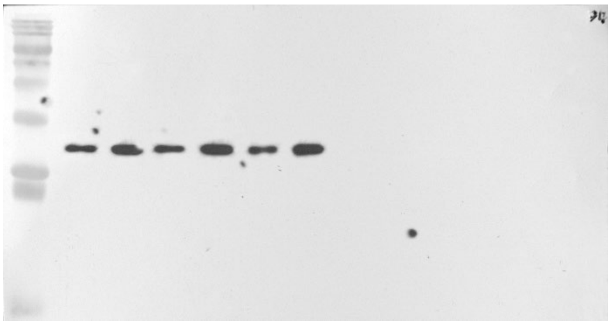

POMC (HFD/HM3D/HFD/HM3D/HFD/HM3D )

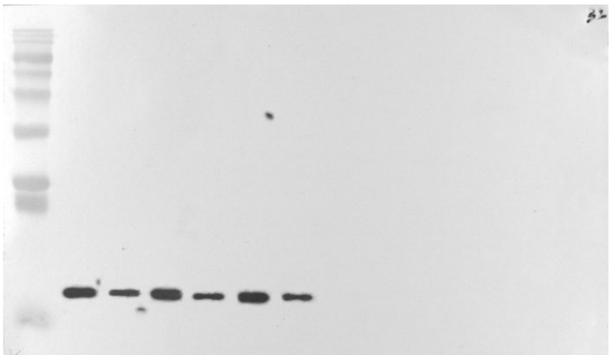

NPY (HFD/HM3D/HFD/HM3D/HFD/HM3D )

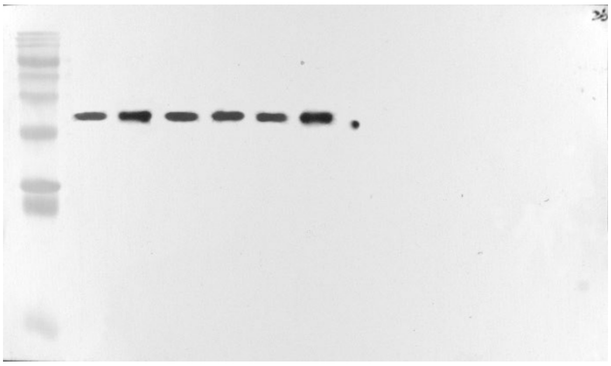

GAPDH (HFD/HM3D/HFD/HM3D/HFD/HM3D )

Quantification

| POMC | | NPY | |
|---|---|---|---|
| **HFD** | **HM3D** | **HFD** | **HM3D** |
| 0.8798 | 1.0482 | 1.3824 | 0.6648 |
| 0.7718 | 1.2356 | 1.2136 | 0.6932 |
| 0.7787 | 0.9629 | 1.3778 | 0.5189 |

## Raw gel images of GLP-1 in figure 3

GLP-1

β-actin

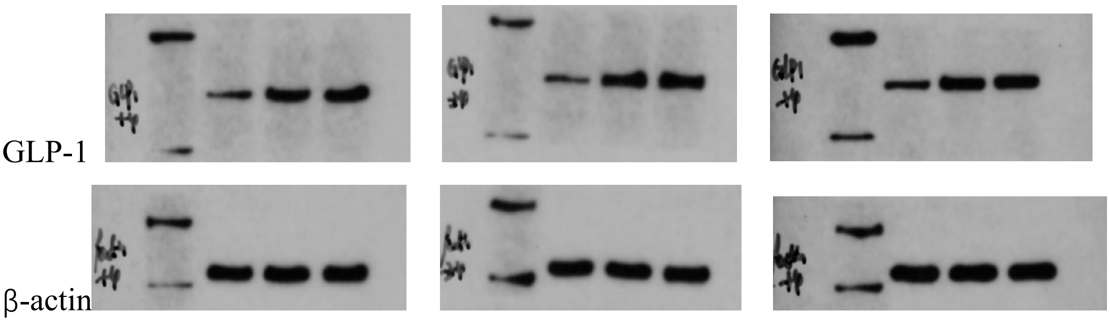

**Quantification**

| HFD | EA | HM3D |
| --- | --- | --- |
| 0.3546 | 0.7596 | 0.8729 |
| 0.2506 | 0.9875 | 1.0147 |
| 0.4574 | 0.8858 | 0.8908 |

**Raw gel images of POMC and NPY in figure 3**

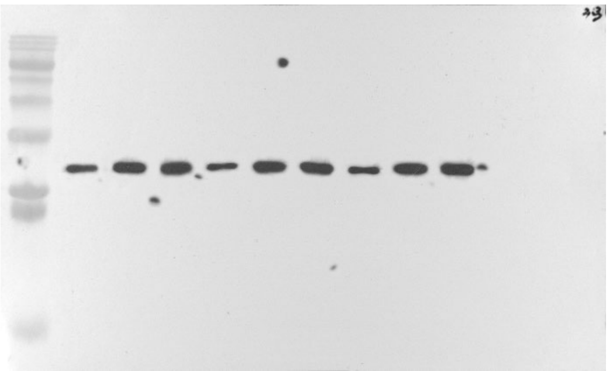

POMC (HFD、EA、HM3D、HFD、EA、HM3D、HFD、EA、HM3D)

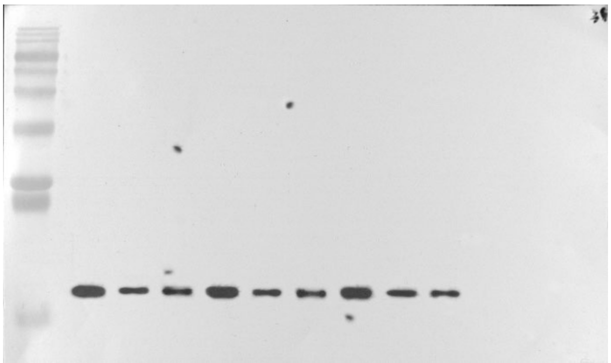

NPY (HFD、EA、HM3D、HFD、EA、HM3D、HFD、EA、HM3D)

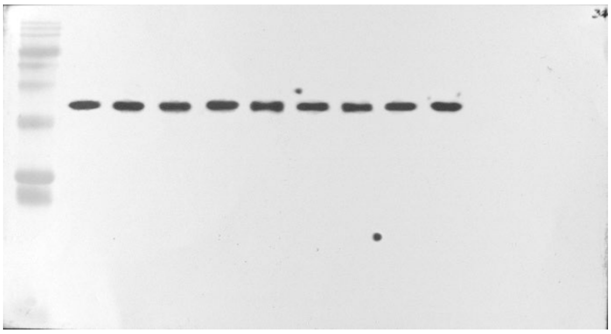

GAPDH (HFD、EA、HM3D、HFD、EA、HM3D、HFD、EA、HM3D)

**Quantification**

| POMC | | | NPY | | |
|---|---|---|---|---|---|
| **HFD** | **EA** | **HM3D** | **HFD** | **EA** | **HM3D** |
| 0.8626 | 1.2921 | 1.3273 | 1.4043 | 0.6625 | 0.8656 |
| 0.6487 | 1.0774 | 1.3575 | 1.1574 | 0.5711 | 0.8001 |
| 0.8714 | 1.5086 | 1.4110 | 1.3276 | 0.8621 | 0.6452 |

**Raw gel images of GLP-1 in figure 4**

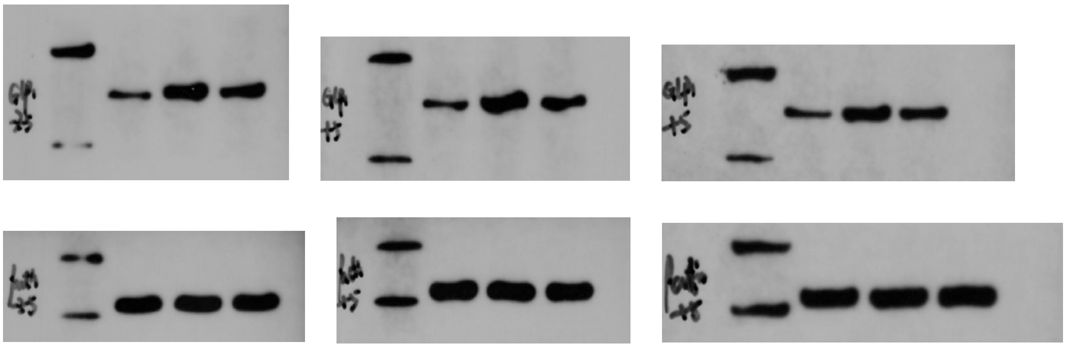

**Quantification**

| HFD | EA | EA+HM4D |
|---|---|---|
| 0.2969 | 0.9516 | 0.7395 |
| 0.3139 | 0.9257 | 0.6238 |
| 0.3711 | 0.8835 | 0.6186 |

**Raw gel images of POMC and NPY in figure 4**

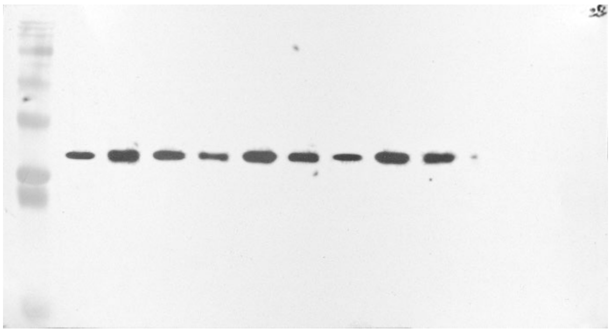

POMC (HFD、EA、EA+HM4D、HFD、EA、EA+HM4D、HFD、EA、EA+HM4D)

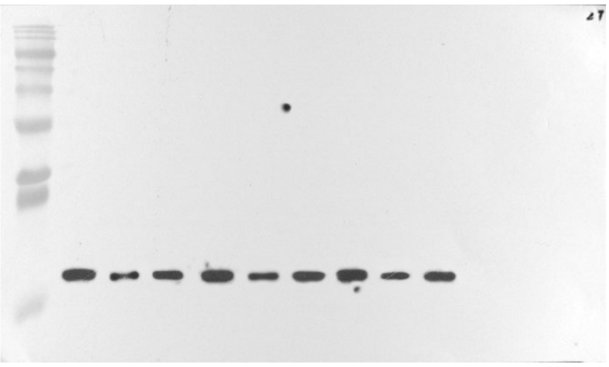

NPY (HFD、EA、EA+HM4D、HFD、EA、EA+HM4D、HFD、EA、EA+HM4D)

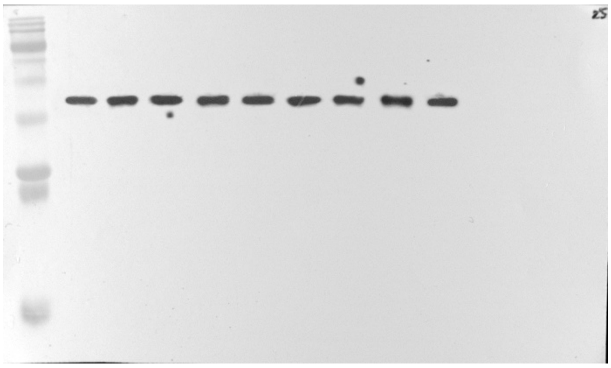

GAPDH (HFD、EA、EA+HM4D、HFD、EA、EA+HM4D、HFD、EA、EA+HM4D)

**Quantification**

| POMC | | | NPY | | |
|---|---|---|---|---|---|
| **HFD** | **EA** | **EA+HM4D** | **HFD** | **EA** | **EA+HM4D** |
| 0.8626 | 1.2921 | 1.3273 | 1.4043 | 0.6625 | 0.8656 |
| 0.6487 | 1.0774 | 1.3575 | 1.1574 | 0.5711 | 0.8001 |
| 0.8714 | 1.5086 | 1.4110 | 1.3276 | 0.8621 | 0.6452 |

## Raw gel images of GLP-1 in figure 5

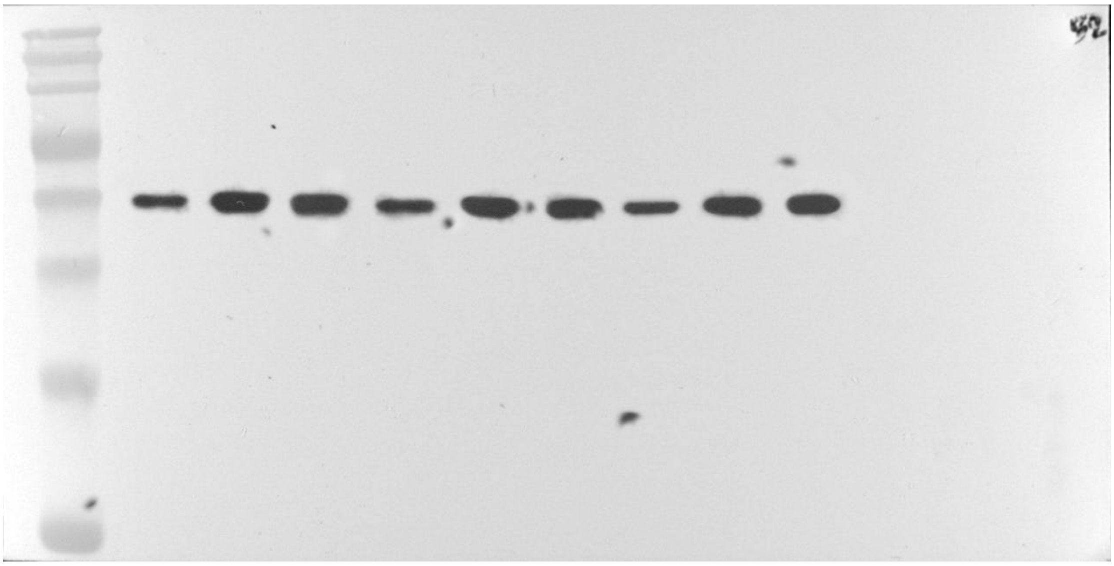

GLP-1 (HFD、EA、taVNS、HFD、EA、taVNS、HFD、EA、taVNS)

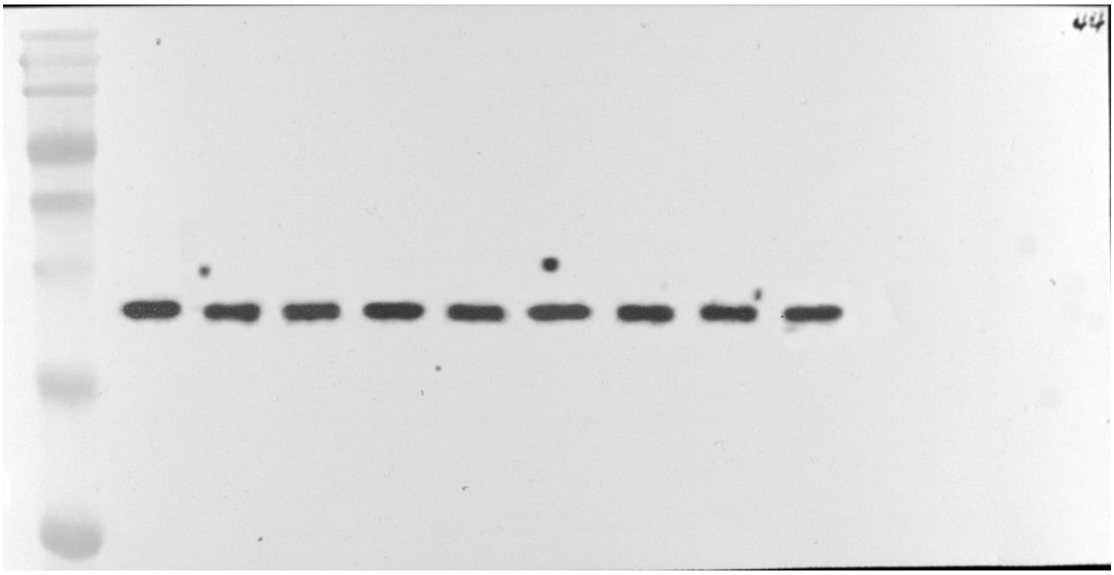

GAPDH (HFD、EA、taVNS、HFD、EA、taVNS、HFD、EA、taVNS)

**Quantification**

| M | EA | taVNS |
|---|---|---|
| 0.7670 | 1.3838 | 1.3713 |
| 0.8134 | 1.2576 | 1.2044 |
| 0.6879 | 1.2080 | 1.1420 |

**Raw gel images of GLP-1 in figure 6**

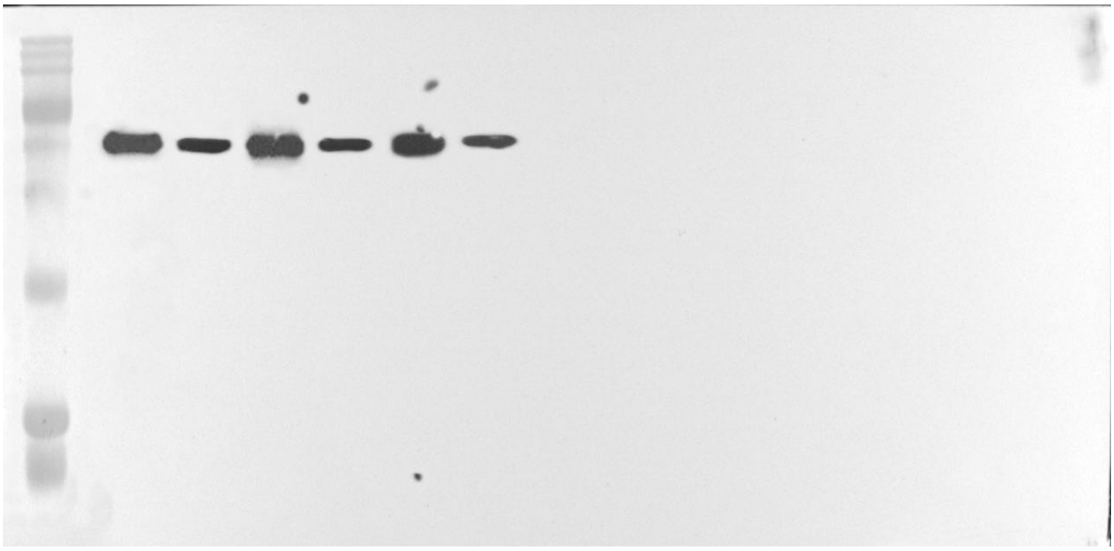

GLP-1 (EA、EA+GVND、EA、EA+GVND、EA、EA+GVND)

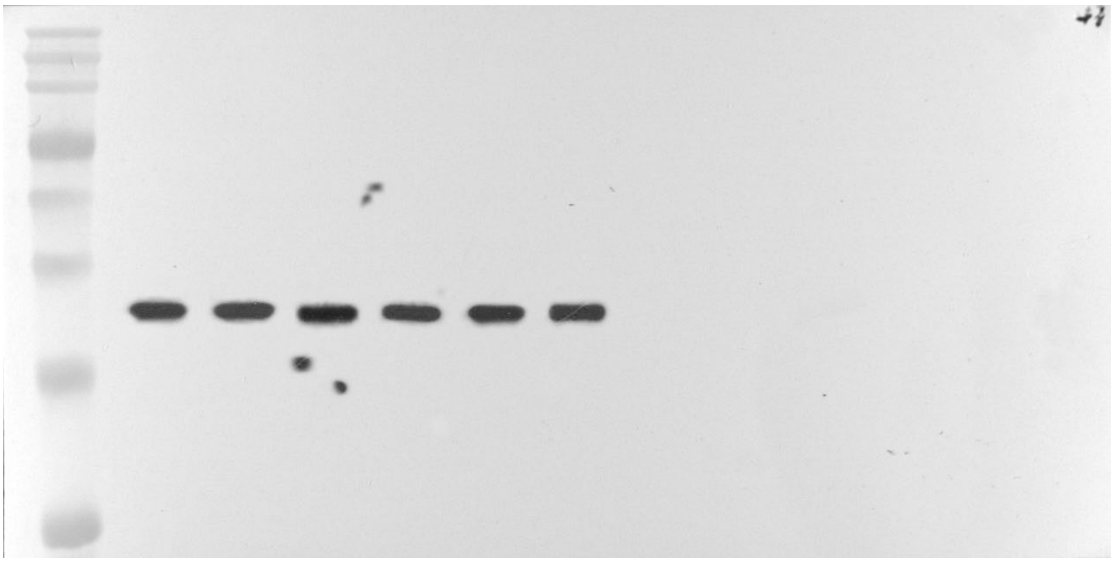

GAPDH (EA、EA+GVND、EA、EA+GVND、EA、EA+GVND)

**Quantification**

| EA | EA+GVND |
|---|---|
| 1.2629 | 0.8206 |
| 1 | 0.7858 |
| 1.4067 | 0.7120 |
