# Supplementary figures and images for: Acupuncture ameliorates diet-induced obesity via the vagal–GLP-1–ARC circuit: neural mechanism of anorexigenic action
Source: Chin Med. 2026 Jan 8;21:20. doi: 10.1186/s13020-025-01274-z (PMC12781781; doi:10.1186/s13020-025-01274-z)

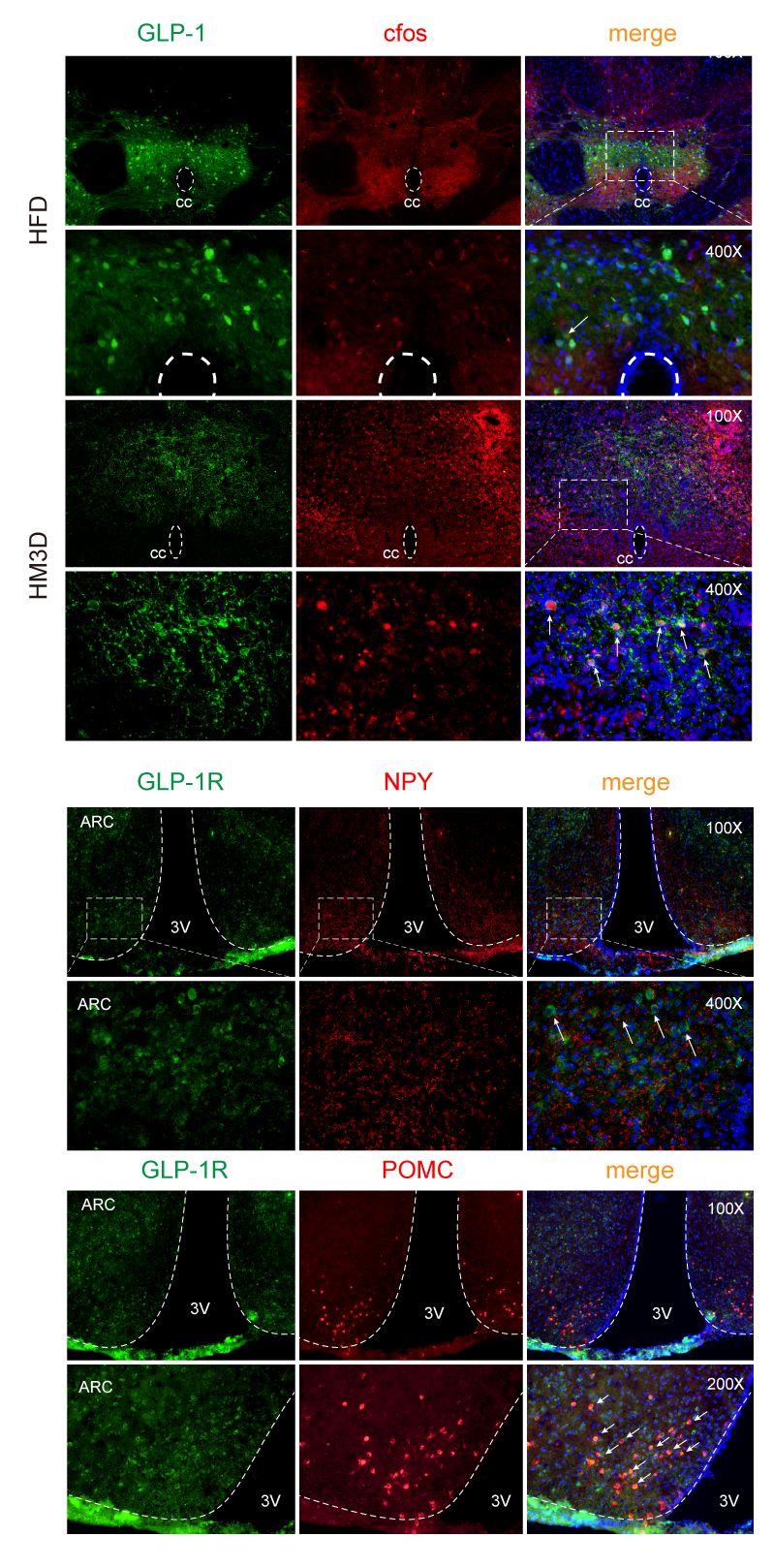

Supplement: Supplementary file 4 — Additional file 4. [file 13020_2025_1274_MOESM4_ESM.tif]

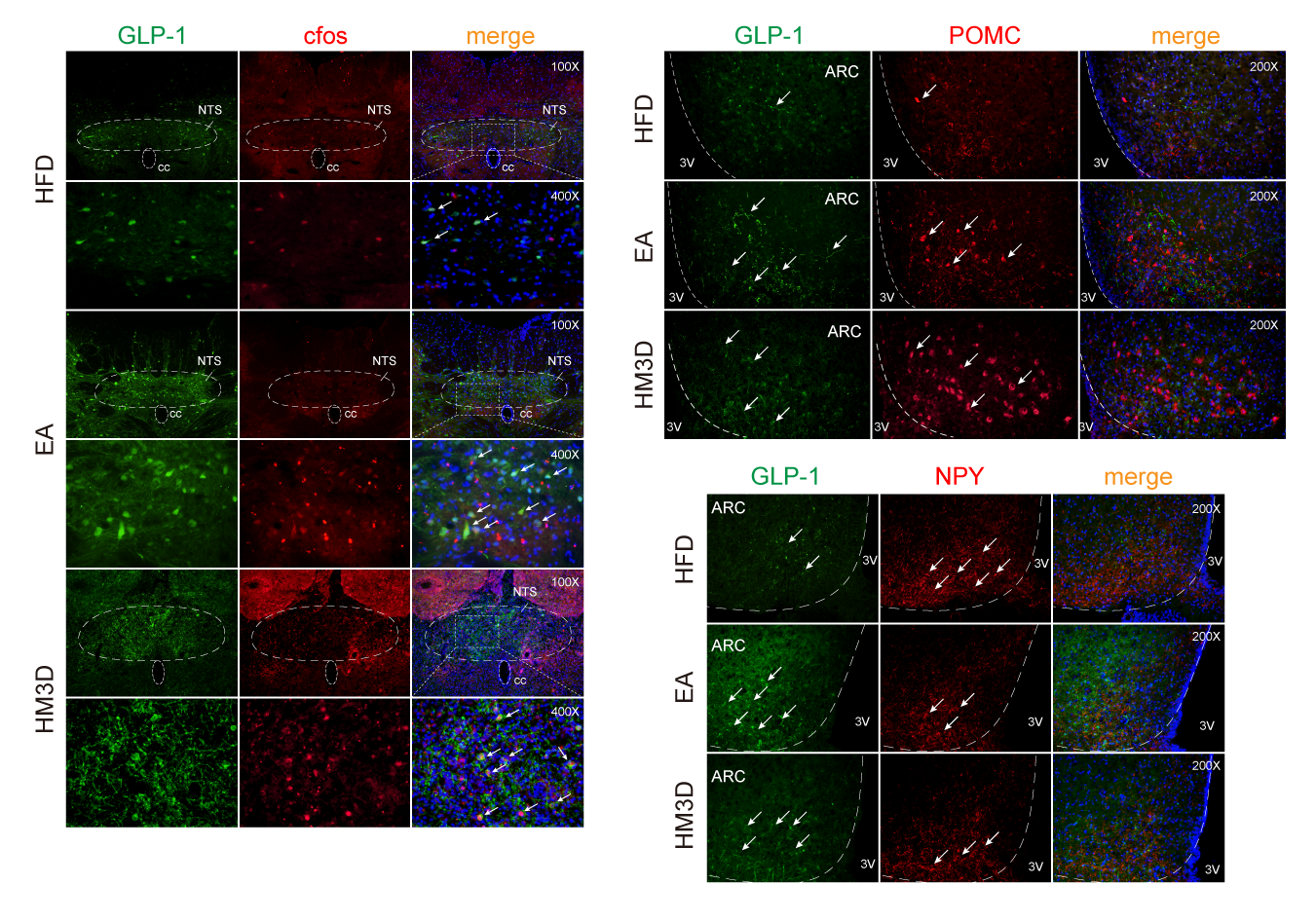

Supplement: Supplementary file 5 — Additional file 5. [file 13020_2025_1274_MOESM5_ESM.tif]

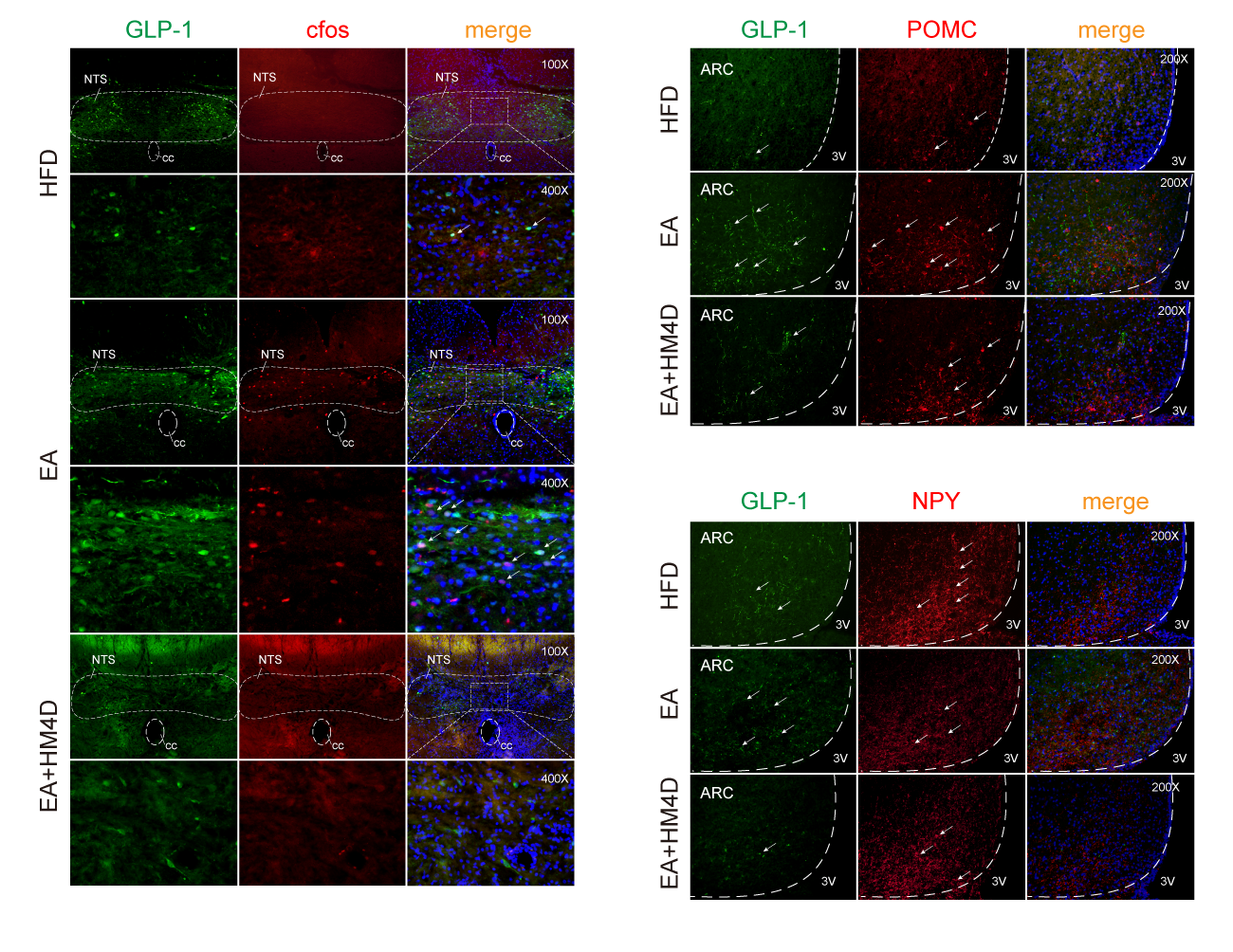

Supplement: Supplementary file 6 — Additional file 6. [file 13020_2025_1274_MOESM6_ESM.tif]

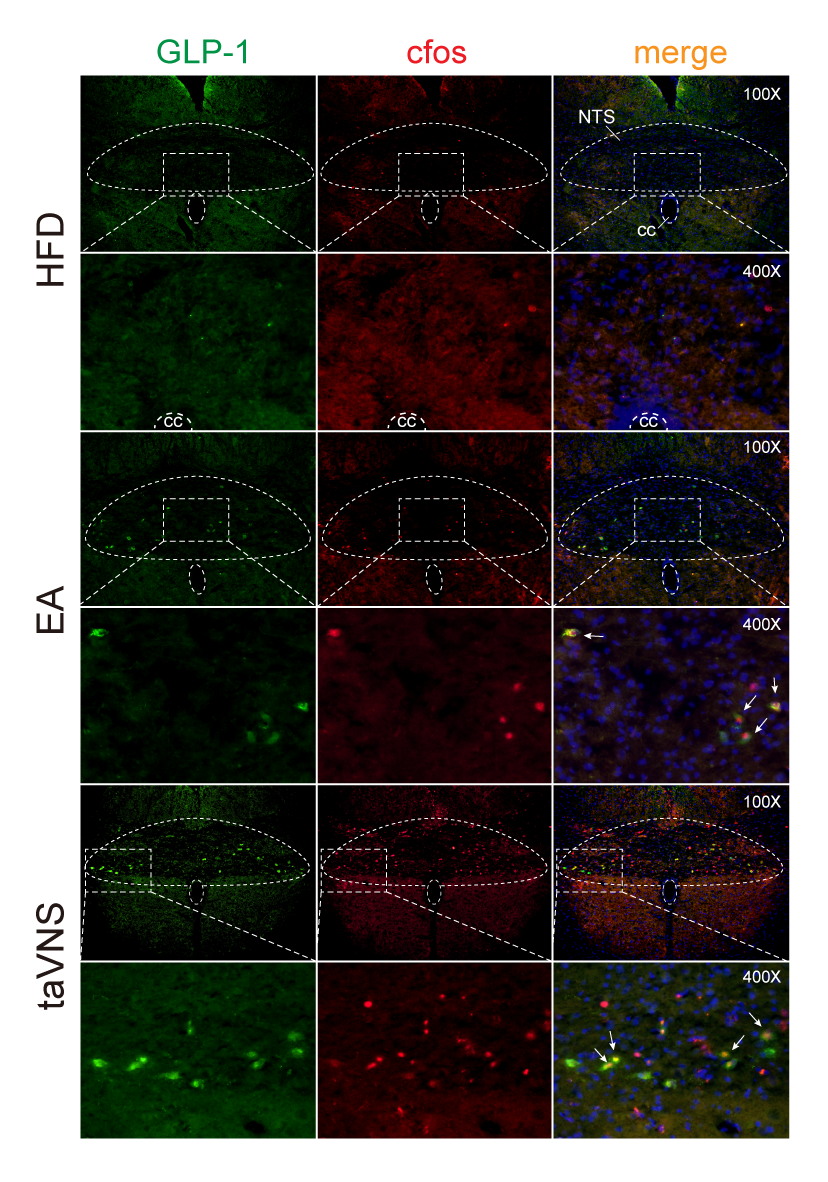

Supplement: Supplementary file 7 — Additional file 7. [file 13020_2025_1274_MOESM7_ESM.tif]

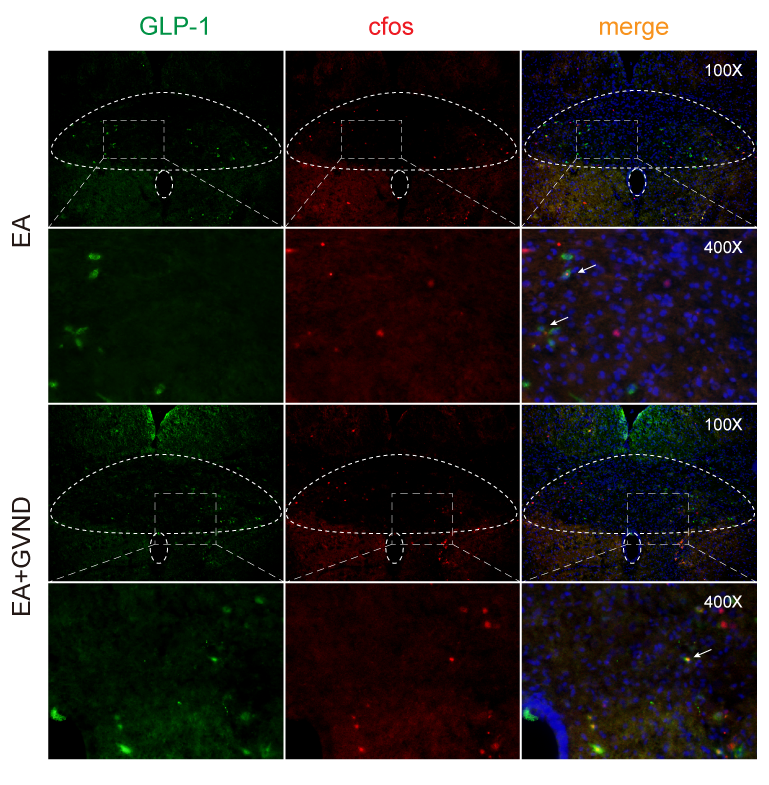

Supplement: Supplementary file 8 — Additional file 8. [file 13020_2025_1274_MOESM8_ESM.tif]

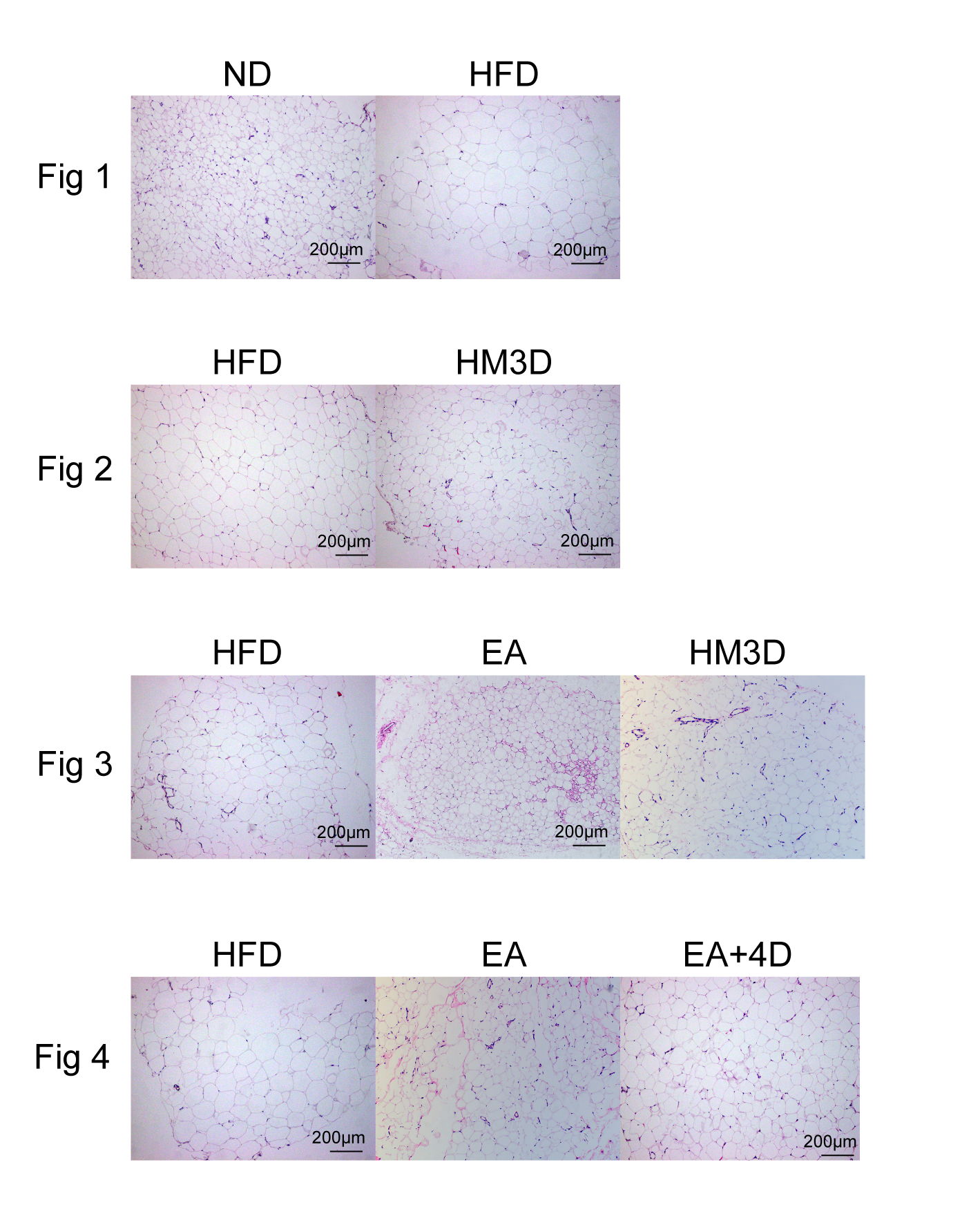

Supplement: Supplementary file 9 — Additional file 9. [file 13020_2025_1274_MOESM9_ESM.tif]
