# Supplementary material for: Acupuncture ameliorates diet-induced obesity via the vagal–GLP-1–ARC circuit: neural mechanism of anorexigenic action
Source: Chin Med. 2026 Jan 8;21:20. doi: 10.1186/s13020-025-01274-z (PMC12781781; doi:10.1186/s13020-025-01274-z)
Supplement: Supplementary file 1 — Additional file 1. [file 13020_2025_1274_MOESM1_ESM.pdf]

武汉大学动物实验中心
动物生物安全三级实验室
Wuhan University Center for Animal Experiment/ABSL-III Laboratory

Dear Dr. Yuwei Shao

The Institutional Animal Care and Use Committee (IACUC) at Center for Animal Experiment, Wuhan University approved your protocol on **3-Nov-2020**.

Project: Effectiveness and Mechanisms of Acupuncture in Suppressing Food Intake and Improving Obesity through "Vagal Afferent Neurons—Nucleus of the Solitary Tract GLP-1--Hypothalamus" Neural Circuits of Appetite

AUP Number: WP2020-08085

Approval Date: 3-Nov-2020.

Expiration Date: 3-Nov-2021.

NOTE: Request for Annual Renewals of ACUPS must be received within the IACUC office 10 days prior to its renewal date.

*Routine Reviews should be submitted at least 6 weeks prior to the scheduled expiration date in order to allow enough time for the review process. Failure to re-submit this AUP for its 3-year review will result in automatic closure on the scheduled expiration date.

Although the IACUC office will make every effort to provide 30 day notice regarding required AUP reviews, investigators must satisfy the above requirements.

In the meantime, should you have any questions regarding this notice, please do not hesitate to contact the IACUC office (027-6875-8757).

If you need original approval document, please contact XXXX at the IACUC office (027-6875-8757).

Thank you for your cooperation with the IACUC.

Sincerely Yours,

IACUC（Seal）

武汉大学动物实验中心 实验动物管理与使用委员会

湖北省武汉市武昌区东湖路 115 号，430071
115 Donghu Road, Wuchang District, Wuhan, Hubei 430071, P.R.China

电话：(+86)27-68758702
Tel: (+86)27-68758702

传真:(+86)27-68758702
Fax: (+86)27-68758702
